# Supplementary material for: Group arts therapies for patients with schizophrenia: a protocol of systematic review and meta-analysis
Source: BMJ Open. 2024 Jun 4;14(6):e082076. doi: 10.1136/bmjopen-2023-082076 (PMC11163625; doi:10.1136/bmjopen-2023-082076)
Supplement: Supplementary data [file bmjopen-2023-082076supp001.pdf]

BMJ Publishing Group Limited (BMJ) disclaims all liability and responsibility arising from any reliance placed on this supplemental material which has been supplied by the author(s)

Group Arts Therapies for Patients with Schizophrenia: A Protocol of Systematic Review and Meta-analysis

Appendix 1. Full search strategies for all databases.

Database	Search strategy
PubMed	((((((((((((((group arts therapies[Title/Abstract]) OR (group painting therapy[Title/Abstract])) OR (group music therapy[Title/Abstract])) OR (group dance therapy[Title/Abstract])) OR (group yoga therapy[Title/Abstract])) OR (group drama therapy[Title/Abstract])) OR (group handicrafts therapy[Title/Abstract])) OR (group collage therapy[Title/Abstract])) OR (group play therapy[Title/Abstract])) OR (group photography therapy[Title/Abstract])) OR (group movie therapy[Title/Abstract])) AND (Schizophrenia, schizophrenias[Title/Abstract])) OR (Schizophrenic Disorders[Title/Abstract])) OR (Schizophrenic Disorder[Title/Abstract])) OR (Disorder, Schizophrenic[Title/Abstract])) OR (Disorders, Schizophrenic[Title/Abstract])
Web of Science	(ALL=(Group Arts therapies)) OR ALL=(Group Painting Therapy)) OR ALL=(Group Music Therapy)) OR ALL=(Group Dance Therapy)) OR ALL=(Group Yoga Therapy)) OR ALL=(Group Drama Therapy)) OR ALL=(Group Handicrafts Therapy)) OR ALL=(Group Collage Therapy)) OR ALL=(Group Play Therapy)) OR ALL=(Group Photography Therapy)) OR ALL=(Group Movie Therapy)) AND ALL=(Schizophrenia, schizophrenias)) OR ALL=(Schizophrenic Disorders)) OR ALL=(Schizophrenic Disorder)) OR ALL=(Disorder, Schizophrenic)) OR ALL=(Disorders, Schizophrenic)
Cochrane Library	Number — Search terms<br>#1 Group Arts therapies<br>#2 Group Painting Therapy<br>#3 Group Music Therapy<br>#4 Group Dance Therapy<br>#5 Group Yoga Therapy<br>#6 Group Drama Therapy<br>#7 Group Handicrafts Therapy<br>#8 Group Collage Therapy<br>#9 Group Play Therapy<br>#10 Group Photography Therapy<br>#11 Group Movie Therapy<br>#12 #1 OR #2 OR #3 OR #4 OR #5 OR #6 OR #7 OR #8 OR #9 OR #10 OR #11<br>#13 Schizophrenia, schizophrenias<br>#14 Schizophrenic Disorders<br>#15 Schizophrenic Disorder<br>#16 Disorder, Schizophrenic<br>#17 Disorders, Schizophrenic<br>#18 #13 OR #14 OR #15 OR #16 OR #17<br>#20 #12 AND #18
Embase	('group arts therapies' OR (('group'/exp OR group) AND arts AND therapies) OR 'group painting therapy' OR (('group'/exp OR group) AND

	('painting'/exp OR painting) AND ('therapy'/exp OR therapy)) OR 'group music therapy' OR (('group'/exp OR group) AND ('music'/exp OR music) AND ('therapy'/exp OR therapy)) OR 'group dance therapy' OR (('group'/exp OR group) AND ('dance'/exp OR dance) AND ('therapy'/exp OR therapy)) OR 'group yoga therapy' OR (('group'/exp OR group) AND ('yoga'/exp OR yoga) AND ('therapy'/exp OR therapy)) OR 'group drama therapy' OR (('group'/exp OR group) AND ('drama'/exp OR drama) AND ('therapy'/exp OR therapy)) OR 'group handicrafts therapy' OR (('group'/exp OR group) AND handicrafts AND ('therapy'/exp OR therapy)) OR 'group collage therapy' OR (('group'/exp OR group) AND collage AND ('therapy'/exp OR therapy)) OR 'group play therapy' OR (('group'/exp OR group) AND ('play'/exp OR play) AND ('therapy'/exp OR therapy)) OR 'group photography therapy' OR (('group'/exp OR group) AND ('photography'/exp OR photography) AND ('therapy'/exp OR therapy)) OR 'group movie therapy' OR (('group'/exp OR group) AND ('movie'/exp OR movie) AND ('therapy'/exp OR therapy))) AND ('schizophrenia, schizophrenias' OR (('schizophrenia,'/exp OR schizophrenia,) AND schizophrenias) OR 'schizophrenic disorders' OR (('schizophrenic'/exp OR schizophrenic) AND ('disorders'/exp OR disorders)) OR 'schizophrenic disorder' OR (('schizophrenic'/exp OR schizophrenic) AND ('disorder'/exp OR disorder)) OR 'disorder, schizophrenic' OR (('disorder,'/exp OR disorder,) AND ('schizophrenic'/exp OR schizophrenic)) OR 'disorders, schizophrenic' OR (('disorders,'/exp OR disorders,) AND ('schizophrenic'/exp OR schizophrenic)))
China National Knowledge Infrastructure (CNKI)	（团体艺术治疗 OR 团体绘画疗法 OR 团体音乐疗法 OR 团体舞蹈疗法 OR 团体瑜伽疗法 OR 团体戏剧疗法 OR 团体手工艺疗法 OR 团体拼贴画疗法 OR 团体游戏疗法 OR 团体摄影疗法 OR 团体电影疗法） AND （精神分裂症 OR 精神分裂）
Wanfang Data	（团体艺术治疗 或 团体绘画疗法 或 团体音乐疗法 或 团体舞蹈疗法 或 团体瑜伽疗法 或 团体戏剧疗法 或 团体手工艺疗法 或 团体拼贴画疗法 或 团体游戏疗法 或 团体摄影疗法 或 团体电影疗法） 与 （精神分裂症 或 精神分裂）
RISS	(((((전체=(집단 예술 치료) OR 전체=(집단 미술 치료) OR 전체=(집단 음악 치료) OR 전체=(집단 무용 치료) OR 전체=(집단 요가 치료) OR 전체=(집단 드라마 치료) OR 전체=(집단 수공예 치료) OR 전체=(집단 콜라주 치료) OR 전체=(집단 놀이 치료) OR 전체=(집단 사진 치료) OR 전체=(집단 영화 치료)) AND 전체=(조현병)) OR 전체=(정신분열증))
Korean Citation Index (KCI)	(((((전체=(집단 예술 치료) OR 전체=(집단 미술 치료) OR 전체=(집단 음악 치료) OR 전체=(집단 무용 치료) OR 전체=(집단 요가 치료) OR 전체=(집단 드라마 치료) OR 전체=(집단 수공예 치료) OR 전체=(집단 콜라주 치료) OR 전체=(집단 놀이 치료) OR 전체=(집단 사진 치료) OR 전체=(집단 영화 치료)) AND 전체=(조현병)) OR 전체=(정신분열증))

DBpia	(((((전체=(집단 예술 치료) OR 전체=(집단 미술 치료) OR 전체=(집단 음악 치료) OR 전체=(집단 무용 치료) OR 전체=(집단 요가 치료) OR 전체=(집단 드라마 치료) OR 전체=(집단 수공예 치료) OR 전체=(집단 콜라주 치료) OR 전체=(집단 놀이 치료) OR 전체=(집단 사진 치료) OR 전체=(집단 영화 치료)) AND 전체=(조현병)) OR 전체=(정신분열증))
