# Supplementary material for: Group arts therapies for patients with schizophrenia: a protocol of systematic review and meta-analysis
Source: BMJ Open. 2024 Jun 4;14(6):e082076. doi: 10.1136/bmjopen-2023-082076 (PMC11163625; doi:10.1136/bmjopen-2023-082076)
Supplement: Author's manuscript [file bmjopen-2023-082076.draft_revisions.pdf]

BMJ Open is committed to open peer review. As part of this commitment we make the peer review history of every article we publish publicly available.

When an article is published we post the peer reviewers' comments and the authors' responses online. We also post the versions of the paper that were used during peer review. These are the versions that the peer review comments apply to.

The versions of the paper that follow are the versions that were submitted during the peer review process. They are not the versions of record or the final published versions. They should not be cited or distributed as the published version of this manuscript.

BMJ Open is an open access journal and the full, final, typeset and author-corrected version of record of the manuscript is available on our site with no access controls, subscription charges or pay-per-view fees (http://bmjopen.bmj.com).

If you have any questions on BMJ Open's open peer review process please email info.bmjopen@bmj.com

Group Arts Therapies for Patients with Schizophrenia: A Protocol of Systematic Review and Meta-analysis

Journal:	*BMJ Open*
Manuscript ID	bmjopen-2023-082076
Article Type:	Protocol
Date Submitted by the Author:	14-Nov-2023

Complete List of Authors:	Zhang, Aijia; Macau University of Science and Technology, Faculty of Humanities and Arts<br>Luo, Xuexing; Macau University of Science and Technology<br>Lin, Runqing; Macau University of Science and Technology<br>He, Caihong; Guangzhou Wanqu Cooperative Institute of Design<br>Wang, Jue; Macau University of Science and Technology; Macau University of Science and Technology<br>Huang, Guanghui; Macau University of Science and Technology; Zhuhai MUST Science and Technology Research Institute

Keywords:	Physiology < NATURAL SCIENCE DISCIPLINES, Meta-Analysis, Schizophrenia & psychotic disorders < PSYCHIATRY

Group Arts Therapies for Patients with Schizophrenia: A Protocol of Systematic Review and Meta-analysis

Abstract

Introduction: Schizophrenia is a chronic mental disorder that profoundly affects cognition, emotion, and social functioning. Given the limitations of conventional pharmacotherapy, such as a plethora of side effects, poor medication adherence, and high costs, group arts therapies (GATs) offers a potential for creative adjunctive treatment to alleviate the symptoms in individuals with schizophrenia. However, the efficacy and safety of GATs remain uncertain. Therefore, this study examines the potential benefits of all group-based artistic interventions as adjunctive treatments for schizophrenia, aiming to systematically evaluate the therapeutic effect of GATs on patients with this condition.

Methods and analysis: Four English databases, two Chinese databases and three Korean databases will be searched from their inception to October 2023: PubMed, Web of Science, Cochrane Library, Embase, Wanfang, China National Knowledge Infrastructure (CNKI), RISS, Korean Citation Index (KCI), and DBpia. All randomized controlled trials comparing GATs for schizophrenia with conventional rehabilitation methods will be included for assessment. Improvement in patients' positive and negative symptoms will be defined as the primary outcome of the study. Bias risk assessment, data synthesis, sensitivity analysis, and subgroup analysis will be conducted using Review Manager 5.4. Study results with low heterogeneity will be combined using a fixed-effect model for meta-analysis ($I^2 < 50\%$ or $p > 0.1$). Conversely, results with high heterogeneity will be merged using a random-effects model ($I^2 > 50\%$ or $p < 0.01$). If a meta-analysis is not feasible due to significant clinical and methodological heterogeneity, a qualitative summary of the findings will be provided.

Ethics and dissemination: All data employed in this systematic review are anonymized and do not involve any private information; consequently, ethical approval is not required. The findings of the research will be disseminated through peer-reviewed publications.

Strengths and limitations of this study

- This represents the first global systematic review and meta-analysis to scrutinize the effects of GATs on schizophrenia, with the outcomes set to further elucidate the

effectiveness of GATs in the treatment of schizophrenia, thereby supplying physicians and patients with additional clinical evidence.

- The study displays relatively minor language bias, as research published in Chinese and Korean has been included alongside English-language studies.
- Potential publication bias may exist due to the exclusion of conference papers, unpublished documents, and ongoing clinical trials from the included literature.
- Significant heterogeneity might be present owing to variations in the type of GAT, as well as differences in the duration and frequency of the treatment.

Introduction

Individuals afflicted with schizophrenia experience a profound compound strain both physiologically and psychologically. A recent epidemiological survey shows that these individuals are at an elevated risk for suicide and unemployment compared with the general populace (1,2). This is because schizophrenia is accompanied by positive symptoms such as hallucinations, delusions, and disordered thinking (3). Physiological pain exacerbates patients' levels of anxiety and depression, leading them to make extreme choices (4). Additionally, negative symptoms like cognitive impairments, emotional flatness, social withdrawal, and a lack of motivation, strip many sufferers of the faculties necessary for engaging in social life. These symptoms are the primary causes for their long-term incidence rate and difficulty in working and living as ordinary people (5-7).

At present, pharmacotherapy serves as the principal treatment approach for schizophrenia, showing relative efficacy in mitigating positive symptoms and preventing relapse (8,9). However, studies suggest that medication alone has limited effectiveness in reducing negative symptoms (10). Furthermore, the dire side effects of long-term medication use, poor patient compliance, and the prohibitive costs of treatment present significant challenges for many patients and their families (11-15). Thus, it is imperative to offer an adjunctive therapeutic option from the perspective of psychotherapy to assist individuals with schizophrenia in their gradual integration into normal life.

Group arts therapies (GATs), a supportive psychotherapeutic approach conducted under the guidance of professional therapists and involving artistic creation with group members, utilizes mediums such as painting, music, dance, and play as healing tools. This method steers patients through creative activities within a group setting to enhance their mental health (16). Certain studies affirm the significant impact of GATs on the social functioning deficits, sense of pleasure, self-efficacy, and interpersonal

relationships of individuals with schizophrenia (17-20). The therapeutic benefits stem partly from the diverse artistic mediums that redirect patients' focus to the process of creation, helping to alleviate anxiety and stress, thus facilitating emotional expression and ameliorating the presentation of positive symptoms (21). Furthermore, by nurturing and fostering the artistic talents of patients, GATs enhances self-confidence and self-esteem, which in turn, improves emotional, cognitive, and social functioning (22). Additionally, the interactive dynamics between patients, therapists, and fellow participants during the therapy support and foster improvement in physiological, psychological, and social well-being (23). For patients with schizophrenia who face language challenges, the use of artistic mediums in a group context can facilitate unobstructed emotional expression, which might be more challenging in traditional, conversation-based interventions (24). By listening to others share their artworks and receiving empathy and encouragement, patients may gain community support, which can aid in alleviating negative symptoms (25,26).

In light of these considerations, GATs holds significant clinical value for enhancing the mental health levels of patients, fostering their recovery, and aiding their gradual reintegration into everyday life. Nonetheless, there has not yet been a systematic evaluation of the effectiveness of GATs for individuals with schizophrenia. Therefore, this review aims to assess the evidence for the efficacy of group art interventions in the clinical treatment of schizophrenia and provide a comparative perspective absent in existing reviews.

Methods

Criteria for including studies in the review

Types of studies

All randomized controlled trials focused on patients diagnosed with schizophrenia will be included in the study. Scholarly journals published in Chinese, English, and Korean will constitute the primary publication types considered for inclusion. Duplicated publications, unpublished data, studies with inaccessible complete information, non-experimental reports, observational studies, and conference papers, literature reviews, case study reports, lectures, or critiques involving outcomes unrelated to GATs interventions will be excluded from this research.

Types of participants

Participants aged 18 to 70 years old, regardless of gender, ethnicity, nationality, educational level, or economic status, will be incorporated into the study. Diagnoses will be based on the Diagnostic and Statistical Manual of Mental Disorders (DSM)-III,

DSM-III-R, DSM-IV, International Classification of Diseases (ICD)-10 criteria, or any other recognized standards. Individuals with significant organic injury, functional impairments, coexisting psychiatric conditions, or those who are unable to continue observation due to severe physical illness, as well as participants with incomplete clinical data, will be excluded from the study.

Types of interventions

Experiments comparing group-based arts therapies interventions with no treatment, standard pharmacotherapy, routine care, or any other form of active psychological counseling intervention will be included. Types of GATs encompass group art therapy, group painting therapy, group music therapy, group dance therapy, or group play therapy. Additionally, studies examining combined interventions, such as GATs in conjunction with standard pharmacotherapy, compared to solely utilizing an alternative modality, such as standard pharmacotherapy alone, also fall within the scope of this research. Given the focus on arts therapies methods conducted within a group or community setting, individualized intervention measures will be excluded.

Types of outcomes

The primary outcomes will be the improvement of positive and negative symptoms in individuals with schizophrenia, to be measured using validated instruments such as the Positive and Negative Syndrome Scale (PANSS).

Secondary outcomes will encompass functional improvements associated with group arts therapies, including assessments of daily living capabilities, social functioning, emotional functioning, and cognitive functioning, as well as adverse events, compliance, costs, and cost-effectiveness. These outcomes will be evaluated using publicly recognized scales such as the Generic Quality of Life Inventory-74 (GQOLI-74), Personal and Social Performance scale (PSP), Nurses' Observation Scale for Inpatient Evaluation (NOSIE), and Tennessee Self-Concept Scale (TSCS), among others.

Electronic searches

Two researchers will conduct a search for all relevant literature published up to October 2023 in English, Chinese, and Korean. The literature will be sourced from databases such as PubMed, Web of Science, Cochrane Library, Embase, Wanfang Data, China National Knowledge Infrastructure (CNKI), RISS, Korean Citation Index (KCI), and DBpia. The keyword combinations used will include: (a) group arts therapies, group painting therapy, group music therapy, group dance therapy, group play therapy, and (b) schizophrenia. The search strategy for PubMed is illustrated in Table 1.

Table 1. Search strategies for English databases, Chinese databases and Korean databases.

Number	Search terms
#1	Group Arts therapies
#2	Group Painting Therapy
#3	Group Music Therapy
#4	Group Dance Therapy
#5	Group Play Therapy
#6	#1 OR #2 OR #3 OR #4 OR #5
#7	Schizophrenia, schizophrenias
#8	Schizophrenic Disorders
#9	Schizophrenic Disorder
#10	Disorder, Schizophrenic
#11	Disorders, Schizophrenic
#12	#7 OR #8 OR #9 OR #10 OR #11
#13	#6 AND #12
#14	tuanti yishu zhiliao (Group Arts therapies)
#15	tuanti huihua zhiliao (Group Painting Therapy)
#16	qunhua zhiliao (Group Painting Therapy)
#17	tuanti yinyue zhiliao (Group Music Therapy)
#18	tuanti wudao zhiliao (Group Dance Therapy)
#19	tuanti youxi zhiliao (Group Play Therapy)
#20	#14 OR #15 OR #16 OR #17 OR #18
#21	jingshenfenliezheng (schizophrenia)
#22	sijueshitiaozheng (schizophrenia)
#23	#21 OR #22
#24	#20 AND #23
#25	jipdanyesulchiryo (집단예술치료)
#26	jipdanmisulchiryo (집단미술치료)
#27	jipdaneumakchiryo (집단음악치료)
#28	jipdan muyongchiryo (집단 무용치료)
#29	jipdannolichiryo (집단놀이치료)
#30	#25 OR #26 OR #27 OR #28 OR #29
#31	johyeonbyeong（조현병，調絃病）
#32	jeongsinbunyeoljeung (정신분열증)
#33	#31 OR #32
#34	#30 AND #33

Searching other resources

The reference lists of published relevant academic journals and review articles will also be manually searched to identify any missing literature. Furthermore, clinical guidelines pertaining to the relevant trials and articles of an ambiguous nature will be manually scrutinized to ensure no eligible studies are overlooked.

For peer review only

Data collection

Selection of studies

Two independent researchers will independently review the titles, abstracts, and full texts of the articles retrieved, compiling studies that meet the inclusion criteria. Any disagreements between researchers will be resolved through discussion to reach a consensus, and if necessary, a third independent reviewer will contribute to the final decision. The flowchart of the Preferred Reporting Items for Systematic Reviews and Meta-Analyses (PRISMA) delineates the selection process of the studies (Figure 1).

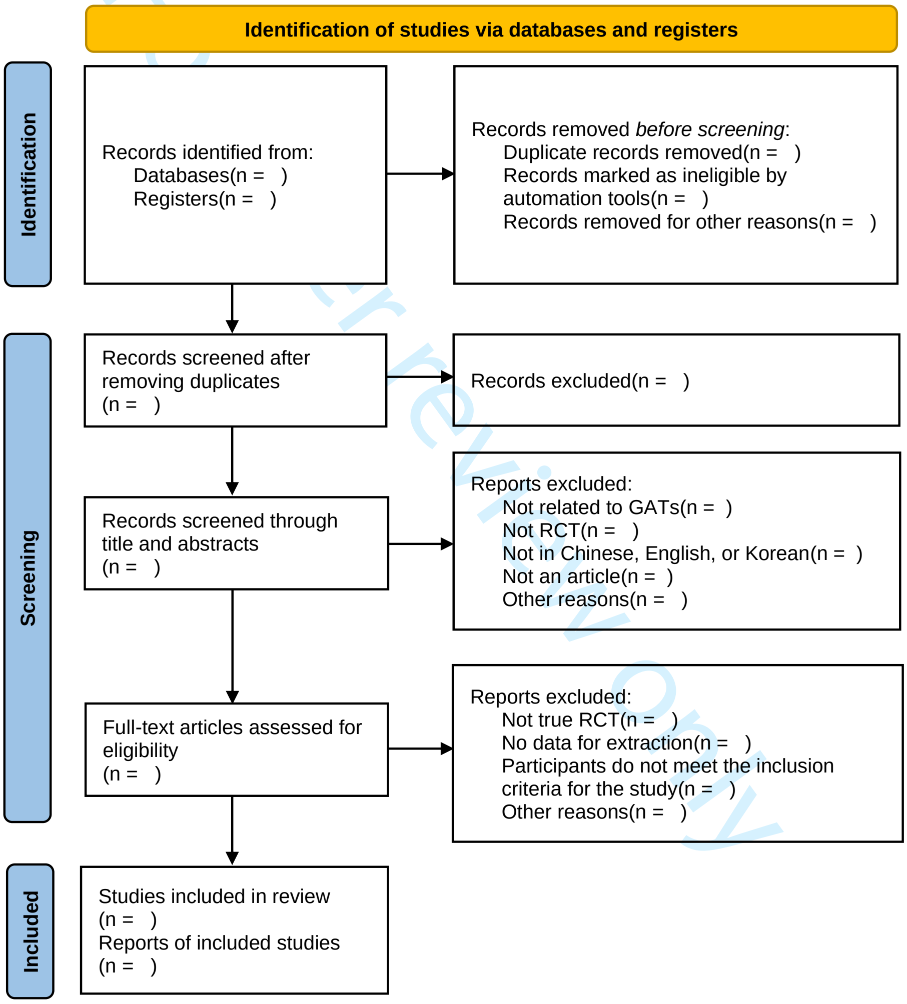

Figure 1. PRISMA flow diagram of the study process. PRISMA, Preferred Reporting Items for Systematic review and Meta-Analysis; RCT, randomized controlled trial; GATs, group arts therapies.

Data extraction and management

Two researchers will independently extract the following information from the included articles using a standardized form: (1) Basic information: first author's name, year of publication, region of publication, number of cases included, type of study design; (2) Clinical and pathological data: age, gender, duration of illness, length of hospital stay; (3) Intervention measures: nature of the intervention, implementer, frequency of intervention per week, duration of the intervention; (4) Outcome measures; (5) Rating scales used. Both researchers will independently extract and code data according to this form. The results of the data extraction will be cross-checked. In case of discrepancies, a third researcher will be involved in the discussion and decision-making process.

Assessment of risk of bias

The risk of bias and methodological quality of all included randomized controlled trials will be assessed by two independent researchers using the Cochrane Risk of Bias 2 (RoB 2) tool (27). Each trial will be evaluated for bias according to the following domains: bias arising from the randomization process, bias due to deviations from the intended interventions, bias due to missing outcome data, bias in measurement of the outcome, and bias in selection of the reported result. Risks of bias will be classified as low, high, or unclear. In cases of disagreement, a third reviewer will be consulted and consensus will be reached through discussion.

Data analysis

Measures of treatment effect

Review Manager (RevMan) 5.4 will be utilized to perform the meta-analysis. Continuous data will be expressed as mean difference (MD) and dichotomous data as risk ratio (RR), both with 95% confidence intervals (CI). When the same outcome is measured in various ways, the standardized mean difference (SMD) with a 95% CI will be chosen to represent continuous data.

Unit of analysis issues

The unit of analysis will be individual participants. Units of outcome measures from different studies will be converted to the International System of Units prior to analysis.

Dealing with missing data

Researchers will attempt to contact the authors included in the study via email to obtain missing or incomplete data. If missing data cannot be obtained, the study will be excluded.

Assessment of heterogeneity

Statistical heterogeneity among the included studies will be assessed using the I^2 statistic and the Chi^2 test. A random-effects model will be employed when there is a likelihood of statistical heterogeneity among the studies ($I^2 > 50\%$ or $p < 0.01$). In cases where there is no statistical heterogeneity ($I^2 < 50\%$ or $p > 0.1$), a fixed-effect model will be utilized for the calculations.

Assessment of reporting biases

Reporting bias will be assessed following the CONSORT guidelines, and a funnel plot will be generated to evaluate potential reporting biases. A symmetric distribution of data within the funnel plot suggests the absence of publication bias. If the funnel plot is asymmetrical, efforts will be made to explore possible explanations beyond publication biases and language biases.

Data synthesis

Data synthesis will be conducted using RevMan 5.4. When two or more studies with low heterogeneity are identified, their results will be combined using a fixed-effect model for the meta-analysis ($I^2 < 50\%$ or $p > 0.1$). Conversely, results of studies with high heterogeneity will be merged using a random-effects model ($I^2 > 50\%$ or $p < 0.01$). In the event that the meta-analysis is impeded by considerable clinical and methodological heterogeneity, a qualitative summary of the results will be provided.

Subgroup analysis and investigation of heterogeneity

In the presence of significant heterogeneity among the included studies, subgroup analyses will be conducted. Subgroups will be formed based on different populations (for example, male versus female), different types of GATs mediums (such as group painting, group music, or group play), and different art therapy settings (such as in hospitals versus at home). The effect estimates for each subgroup will be calculated and compared inter-subgroup.

Sensitivity analysis

To ensure the robustness of the evidence, sensitivity analyses will be performed to assess the impact of studies with a high risk of bias and to explore possible sources of heterogeneity. Decisions to exclude lower-quality studies will be made by comparing outcomes and considering factors such as sample size, strength of evidence, and their influence on the pooled effective sample size.

Evidence quality evaluation

The quality of evidence will be further assessed using the Grading of Recommendations Assessment, Development and Evaluation (GRADE) approach, which categorizes the

quality of evidence into four levels: very low, low, moderate, or high. In interpreting the findings, consideration will also be given to the quality of evidence, the context of the studies, potential benefits and harms, and patient values.

Patient and public involvement

Patients or the public were not involved in the study.

Ethics and dissemination

This systematic review does not require ethical approval, as all data utilized are anonymous and do not involve any private information. The outcomes of the research will offer evidence on the efficacy and safety of GATs as an adjunctive therapeutic approach for schizophrenia, and will be disseminated through peer-reviewed publications.

Discussion

While some explorations of GATs in the field of schizophrenia have been undertaken, evidence of its effectiveness in ameliorating positive, negative, and other related symptoms in patients remains limited. The first systematic MATISSE study indicated that group arts therapies did not improve the overall functioning or mental health of individuals with schizophrenia (28), casting doubt on the overall efficacy of GATs. Since then, researchers have been seeking evidence to support the mechanistic changes induced by GATs. A meta-analysis intended to evaluate the effectiveness of arts therapies for schizophrenia was registered in July 2021, but the applicant failed to provide any study outcomes. Uttley et al. in a systematic review of GATs for patients with non-psychotic mental health disorders suggested that group arts therapies might offer cost-effectiveness, yet there was insufficient evidence for comparison (29). Additionally, whether subgroups based on the type, medium, duration, or frequency of GATs sessions relate to treatment effectiveness remains unknown. Subgroup analyses based on these variables may help address this issue. Therefore, this research aims to systematically evaluate the evidence of the effectiveness of group-based artistic interventions in clinical treatment for schizophrenia to provide a comparative viewpoint not offered by existing reviews. Our study seeks to potentially benefit patients and offer further clinical evidence for health practitioners and patients deciding on the use of GATs as an adjunctive treatment for schizophrenia.

This study also has potential limitations. While efforts were made to include research published in multiple languages (English, Chinese, and Korean), studies in other languages (such as Japanese, Spanish) might have been missed, indicating a possible language bias. Additionally, publication bias may exist due to the exclusion of

conference papers, unpublished literature, and ongoing clinical trials. Furthermore, heterogeneity may arise from differences in the type, medium, duration, and frequency of GATs administered. Nonetheless, this systematic approach still contributes to a broader understanding of GATs for schizophrenia.

References

1. Correll, C. U., Solmi, M., Croatto, G., Schneider, L. K., Rohani-Montez, S. C., Fairley, L., ... & Tiihonen, J. (2022). Mortality in people with schizophrenia: a systematic review and meta-analysis of relative risk and aggravating or attenuating factors. World Psychiatry, 21(2), 248-271. doi: 10.1002/wps.20994

2. Crespo-Facorro, B., Such, P., Nylander, A. G., Madera, J., Resemann, H. K., Worthington, E., ... & Newton, R. (2021). The burden of disease in early schizophrenia–a systematic literature review. Current Medical Research and Opinion, 37(1), 109-121. doi: 10.1080/03007995.2020.1841618

3. Andreasen, N. (1995). Symptoms, signs, and diagnosis of schizophrenia. The Lancet, 346(8973), 477-481. doi: 10.1016/S0140-6736(95)91325-4

4. Temmingh, H., & Stein, D. J. (2015). Anxiety in patients with schizophrenia: Epidemiology and management. CNS drugs, 29, 819-832. doi: 10.1007/s40263-015-0282-7

5 Marder, S. R., & Galderisi, S. (2017). The current conceptualization of negative symptoms in schizophrenia. World Psychiatry, 16(1), 14-24. doi: 10.1002/wps.20385

6.. Buchanan, R. W. (2007). Persistent negative symptoms in schizophrenia: an overview. Schizophrenia bulletin, 33(4), 1013-1022. doi: 10.1093/schbul/sbl057

7. Milev, P., Ho, B. C., Arndt, S., & Andreasen, N. C. (2005). Predictive values of neurocognition and negative symptoms on functional outcome in schizophrenia: a longitudinal first-episode study with 7-year follow-up. American Journal of Psychiatry, 162(3), 495-506. doi: 10.1176/appi.ajp.162.3.495

8. Leucht, S., Tardy, M., Komossa, K., Heres, S., Kissling, W., Salanti, G., & Davis, J. M. (2012). Antipsychotic drugs versus placebo for relapse prevention in schizophrenia: a systematic review and meta-analysis. The Lancet, 379(9831), 2063-2071. doi: 10.1016/S0140-6736（12）60239-6

For peer review only

9. Kane, J. M., & Correll, C. U. (2010). Past and present progress in the pharmacologic treatment of schizophrenia. The Journal of clinical psychiatry, 71(9), 13909. doi: 10.4088/JCP.10r06264yel

10. Kirkpatrick, B., Fenton, W. S., Carpenter Jr, W. T., & Marder, S. R. (2006). The NIMH-MATRICS consensus statement on negative symptoms. Schizophrenia bulletin, 32(2), 214-219. doi: 10.1093/schbul/sbj053

11. De las Cuevas, C., Sanz, E. J., & de Leon, J. (2023). Adverse drug reactions and their fatal outcomes in clozapine patients in VigiBase: Comparing the top four reporting countries (US, UK, Canada and Australia). Schizophrenia Research. doi: 10.1016/j.schres.2023.05.004

12. Papola, D., Ostuzzi, G., Gastaldon, C., Morgano, G. P., Dragioti, E., Carvalho, A. F., ... & Barbui, C. (2019). Antipsychotic use and risk of life-threatening medical events: umbrella review of observational studies. Acta Psychiatrica Scandinavica, 140(3), 227-243. doi: 10.1111/acps.13066

13. Lindström, E., & Bingefors, K. (2000). Patient compliance with drug therapy in schizophrenia: economic and clinical issues. Pharmacoeconomics, 18, 105-124. doi: 10.2165/00019053-200018020-00002

14. Peng, M. M., Xing, J., Tang, X., Wu, Q., Wei, D., & Ran, M. S. (2022). Disease-related risk factors for caregiver burden among family caregivers of persons with schizophrenia: a systematic review and meta-analysis. International Journal of Environmental Research and Public Health, 19(3), 1862. doi: 10.3390/ijerph19031862

15. Jin, H., & Mosweu, I. (2017). The societal cost of schizophrenia: a systematic review. Pharmacoeconomics, 35, 25-42. doi: 10.1007/s40273-016-0444-6

16. Karkou V, Sanderson P. (2005). Arts therapies: a research-based map of the field. London: Elsevier Churchill Livingstone.

17. Orfanos, S., Banks, C., & Priebe, S. (2015). Are group psychotherapeutic treatments effective for patients with schizophrenia? A systematic review and meta-analysis. Psychotherapy and psychosomatics, 84(4), 241-249. https://doi.org/10.1159/000377705

18. Cho, J. M., & Lee, K. (2018). Effects of motivation interviewing using a group art therapy program on negative symptoms of schizophrenia. Archives of psychiatric

For peer review only

nursing, 32(6), 878-884. https://doi.org/10.1016/j.apnu.2018.07.002

19. Tong, J., Yu, W., Fan, X., Sun, X., Zhang, J., Zhang, J., & Zhang, T. (2021). Impact of group art therapy using traditional Chinese materials on self-efficacy and social function for individuals diagnosed with schizophrenia. Frontiers in Psychology, 11, 571124. https://doi.org/10.3389/fpsyg.2020.571124

20. Green, B. L., Wehling, C., & Taisky, G. J. (1987). Group art therapy as an adjunct to treatment for chronic outpatients. Psychiatric Services, 38(9), 988-991. https://doi.org/10.1176/ps.38.9.988

21. Waller, D. (2014). Group interactive art therapy: Its use in training and treatment. Routledge.

22. Chandraiah, S., Ainlay Anand, S., & Avent, L. C. (2012). Efficacy of group art therapy on depressive symptoms in adult heterogeneous psychiatric outpatients. Art Therapy, 29(2), 80-86. doi: 10.1080/07421656.2012.683739

23. Waller, D., & Gilroy, A. (2000). Art Therapy: A handbook of new theories and practice. Buckingham: Open University Press.

24. Gabel, A., & Robb, M. (2017). (Re) considering psychological constructs: A thematic synthesis defining five therapeutic factors in group art therapy. The arts in psychotherapy, 55, 126-135. doi: 10.1016/j.aip.2017.05.005

25. Jewell, M., & Camden-Pratt, C. (2023). Creating 'art-alongside'in Peer Art Therapy (PATh) groups: nurturing connection and trust, and responding to power dynamics. International Journal of Art Therapy, 28:1-2, 38-50. doi: 10.1080/17454832.2023.2175002

26. Springham, N., & Camic, P. M. (2017). Observing mentalizing art therapy groups for people diagnosed with borderline personality disorder. International Journal of Art Therapy, 22(3), 138-152. https://doi.org/10.1080/17454832.2017.1288753

27. Higgins, J. P., Sterne, J. A., Savovic, J., Page, M. J., Hróbjartsson, A., Boutron, I., ... & Eldridge, S. (2016). A revised tool for assessing risk of bias in randomized trials. Cochrane database of systematic reviews, 10 (Suppl 1), 29-31. dx.doi.org/10.1002/14651858.CD201601

28. Crawford, M. J., Killaspy, H., Kalaitzaki, E., Barrett, B., Byford, S., Patterson, S., ...

For peer review only

& Waller, D. (2010). The MATISSE study: a randomised trial of group art therapy for people with schizophrenia. BMC psychiatry, 10(1), 1-9. https://doi.org/10.1186/1471-244X-10-65

29. Uttley, L., Stevenson, M., Scope, A., Rawdin, A., & Sutton, A. (2015). The clinical and cost effectiveness of group art therapy for people with non-psychotic mental health disorders: a systematic review and cost-effectiveness analysis. BMC psychiatry, 15(1), 1-13. https://doi.org/10.1186/s12888-015-0528-4

Footnotes

Competing interests: None declared.

Patient and public involvement: Patients and the public were not involved in the design, or conduct, or reporting, or dissemination plans of this research.

Patient consent for publication: Not required.

Provenance and peer review: Not commissioned; externally peer reviewed.

eview only

Table 1. Search strategies for English databases, Chinese databases and Korean databases.

Number	Search terms
#1	Group Arts therapies
#2	Group Painting Therapy
#3	Group Music Therapy
#4	Group Dance Therapy
#5	Group Play Therapy
#6	#1 OR #2 OR #3 OR #4 OR #5
#7	Schizophrenia, schizophrenias
#8	Schizophrenic Disorders
#9	Schizophrenic Disorder
#10	Disorder, Schizophrenic
#11	Disorders, Schizophrenic
#12	#7 OR #8 OR #9 OR #10 OR #11
#13	#6 AND #12
#14	tuanti yishu zhiliao (Group Arts therapies)
#15	tuanti huihua zhiliao (Group Painting Therapy)
#16	qunhua zhiliao (Group Painting Therapy)
#17	tuanti yinyue zhiliao (Group Music Therapy)
#18	tuanti wudao zhiliao (Group Dance Therapy)
#19	tuanti youxi zhiliao (Group Play Therapy)
#20	#14 OR #15 OR #16 OR #17 OR #18
#21	jingshenfenliezheng (schizophrenia)
#22	sijueshitiaozheng (schizophrenia)
#23	#21 OR #22
#24	#20 AND #23
#25	jipdanyesulchiryo (집단예술치료)
#26	jipdanmisulchiryo (집단미술치료)
#27	jipdaneumakchiryo (집단음악치료)
#28	jipdan muyongchiryo (집단 무용치료)
#29	jipdannolichiryo (집단놀이치료)
#30	#25 OR #26 OR #27 OR #28 OR #29
#31	johyeonbyeong（조현병, 調絃病）
#32	jeongsinbunyeoljeung (정신분열증)
#33	#31 OR #32
#34	#30 AND #33

For peer review only

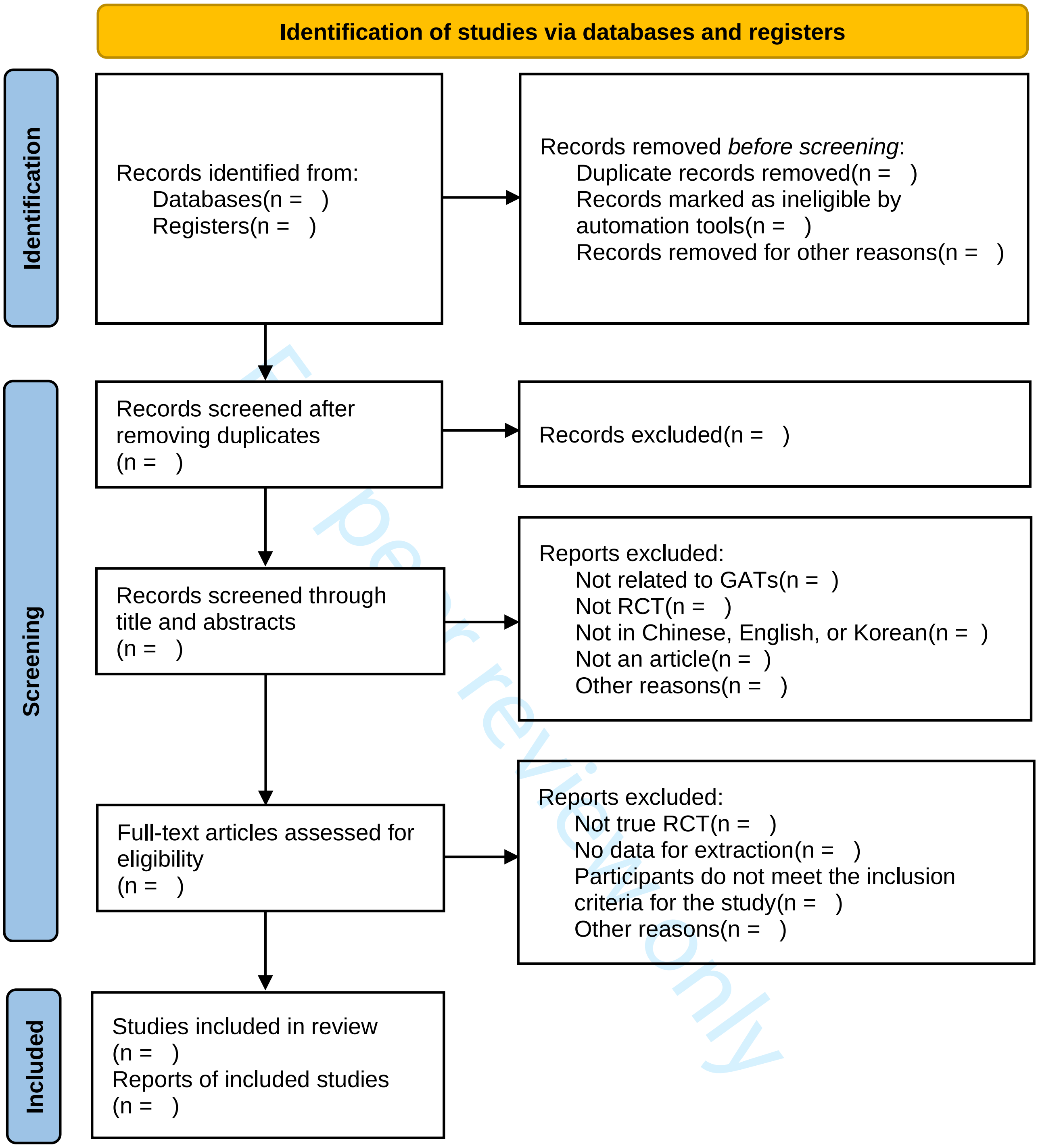

Figure 1. PRISMA flow diagram of the study process. PRISMA, Preferred Reporting Items for Systematic review and Meta-Analysis; RCT, randomized controlled trial; GATs, group arts therapies.

For peer review only - http://bmjopen.bmj.com/site/about/guidelines.xhtml

PRISMA-P (Preferred Reporting Items for Systematic review and Meta-Analysis Protocols) 2015 checklist: recommended items to address in a systematic review protocol*

Section and topic	Item No	Checklist item
**ADMINISTRATIVE INFORMATION**		
√ Title:		
Identification	1a	Identify the report as a protocol of a systematic review
Update	1b	If the protocol is for an update of a previous systematic review, identify as such
√ Registration	2	If registered, provide the name of the registry (such as PROSPERO) and registration number
√ Authors:		
Contact	3a	Provide name, institutional affiliation, e-mail address of all protocol authors; provide physical mailing address of corresponding author
Contributions	3b	Describe contributions of protocol authors and identify the guarantor of the review
√ Amendments	4	If the protocol represents an amendment of a previously completed or published protocol, identify as such and list changes; otherwise, state plan for documenting important protocol amendments
√ Support:		
Sources	5a	Indicate sources of financial or other support for the review
Sponsor	5b	Provide name for the review funder and/or sponsor
Role of sponsor or funder	5c	Describe roles of funder(s), sponsor(s), and/or institution(s), if any, in developing the protocol
√ **INTRODUCTION**		
√ Rationale	6	Describe the rationale for the review in the context of what is already known
√ Objectives	7	Provide an explicit statement of the question(s) the review will address with reference to participants, interventions, comparators, and outcomes (PICO)
**METHODS**		
√ Eligibility criteria	8	Specify the study characteristics (such as PICO, study design, setting, time frame) and report characteristics (such as years considered, language, publication status) to be used as criteria for eligibility for the review
√ Information sources	9	Describe all intended information sources (such as electronic databases, contact with study authors, trial registers or other grey literature sources) with planned dates of coverage
√ Search strategy	10	Present draft of search strategy to be used for at least one electronic database, including planned limits, such that it could be repeated
√ Study records:		
Data management	11a	Describe the mechanism(s) that will be used to manage records and data throughout the review

For peer review only

	Selection process	11b	State the process that will be used for selecting studies (such as two independent reviewers) through each phase of the review (that is, screening, eligibility and inclusion in meta-analysis)
	Data collection process	11c	Describe planned method of extracting data from reports (such as piloting forms, done independently, in duplicate), any processes for obtaining and confirming data from investigators
√	Data items	12	List and define all variables for which data will be sought (such as PICO items, funding sources), any pre-planned data assumptions and simplifications
√	Outcomes and prioritization	13	List and define all outcomes for which data will be sought, including prioritization of main and additional outcomes, with rationale
√	Risk of bias in individual studies	14	Describe anticipated methods for assessing risk of bias of individual studies, including whether this will be done at the outcome or study level, or both; state how this information will be used in data synthesis
√	Data synthesis	15a	Describe criteria under which study data will be quantitatively synthesised
		15b	If data are appropriate for quantitative synthesis, describe planned summary measures, methods of handling data and methods of combining data from studies, including any planned exploration of consistency (such as I^2, Kendall's τ)
		15c	Describe any proposed additional analyses (such as sensitivity or subgroup analyses, meta-regression)
		15d	If quantitative synthesis is not appropriate, describe the type of summary planned
√	Meta-bias(es)	16	Specify any planned assessment of meta-bias(es) (such as publication bias across studies, selective reporting within studies)
√	Confidence in cumulative evidence	17	Describe how the strength of the body of evidence will be assessed (such as GRADE)

*** It is strongly recommended that this checklist be read in conjunction with the PRISMA-P Explanation and Elaboration (cite when available) for important clarification on the items. Amendments to a review protocol should be tracked and dated. The copyright for PRISMA-P (including checklist) is held by the PRISMA-P Group and is distributed under a Creative Commons Attribution Licence 4.0.**

From: Shamseer L, Moher D, Clarke M, Ghersi D, Liberati A, Petticrew M, Shekelle P, Stewart L, PRISMA-P Group. Preferred reporting items for systematic review and meta-analysis protocols (PRISMA-P) 2015: elaboration and explanation. BMJ. 2015 Jan 2;349(jan02 1):g7647.

For peer review only

BMJ Open

Group Arts Therapies for Patients with Schizophrenia: A Protocol of Systematic Review and Meta-analysis

Journal:	*BMJ Open*
Manuscript ID	bmjopen-2023-082076.R1
Article Type:	Protocol
Date Submitted by the Author:	04-Mar-2024
Complete List of Authors:	Zhang, Aijia; Macau University of Science and Technology, Faculty of Humanities and Arts<br>Luo, Xuexing; Macau University of Science and Technology<br>Lin, Runqing; Macau University of Science and Technology<br>He, Caihong; Guangzhou Wanqu Cooperative Institute of Design<br>Wang, Jue; Macau University of Science and Technology, ; Macau University of Science and Technology<br>Huang, Guanghui; Macau University of Science and Technology; Zhuhai MUST Science and Technology Research Institute
**Primary Subject Heading**:	Mental health
Secondary Subject Heading:	Qualitative research
Keywords:	Physiology < NATURAL SCIENCE DISCIPLINES, Meta-Analysis, Schizophrenia & psychotic disorders < PSYCHIATRY

SCHOLARONE™
Manuscripts

Group Arts Therapies for Patients with Schizophrenia: A Protocol of Systematic Review and Meta-analysis

Abstract

Introduction: Schizophrenia, a chronic mental problem, significantly impacts cognition, emotion, and social functioning. Conventional pharmacotherapy faces challenges including numerous side effects, low adherence to medication, and substantial costs. In this context, Group Arts Therapies (GATs) emerge as a promising complementary approach for symptom alleviation in schizophrenia patients. Nonetheless, the effectiveness and safety of GATs are yet to be firmly established. This study aims to systematically assess the therapeutic impact of all group-based artistic interventions as complementary treatments for schizophrenia, focusing on their potential benefits.

Methods and analysis: This study will search four English-language databases (PubMed, Web of Science, Cochrane Library, Embase), two Chinese databases (Wanfang Data, China National Knowledge Infrastructure), and three Korean databases (RISS, Korean Citation Index, DBpia) from their inception until October 2023. It will include all randomized controlled trials (RCTs) that compare Group Arts Therapies (GATs) for schizophrenia with standard rehabilitation methods. The primary outcome is the improvement in patients' positive and negative symptoms. Methodologies such as bias risk assessment, data synthesis, sensitivity analysis, and subgroup analysis will be implemented using Review Manager 5.4. Study results with high heterogeneity will be merged using a random-effects model ($I^2 > 50\%$ or $p < 0.1$). In cases where meta-analysis is not viable due to significant clinical and methodological heterogeneity, a qualitative summary of the findings will be provided.

Ethics and dissemination: The data utilized in this systematic review are anonymized, devoid of any private information, eliminating the requirement for ethical approval. Dissemination of the research findings will be conducted via peer-reviewed publications.

PROSPERO registration number: CRD42023471583.

Strengths and limitations of this study

- This represents the first global systematic review and meta-analysis to scrutinize the effects of GATs on schizophrenia, with the outcomes set to further elucidate the effectiveness of GATs in the treatment of schizophrenia, thereby supplying physicians and patients with additional clinical evidence.
- The study displays relatively minor language bias, as research published in Chinese and Korean has been included alongside English-language studies.
- Potential publication bias may exist due to the exclusion of conference papers, unpublished documents, and ongoing clinical trials from the included literature.
- Significant heterogeneity might be present owing to variations in the type of GAT, as well as differences in the duration and frequency of the treatment.

Introduction

Individuals with schizophrenia endure significant physiological and psychological stress. A recent epidemiological survey shows that these individuals face a higher risk of suicide and unemployment compared to the general population (1,2). This increased risk is attributed to the presence of positive symptoms such as hallucinations, delusions, and disordered thinking (3). Furthermore, physiological distress intensifies feelings of anxiety and depression, potentially leading to dire decisions (4). The condition is also characterized by negative symptoms, including cognitive impairments, emotional flatness, social withdrawal, and diminished motivation, which severely limit the ability to participate in social life. These symptoms are the primary causes for their long-term incidence rate and may struggle to undertake activities of daily living (5-7).

At present, pharmacotherapy serves as the principal treatment approach for schizophrenia, showing relative efficacy in mitigating positive symptoms and preventing relapse (8,9). However, studies suggest that medication alone has limited effectiveness in reducing negative symptoms (10). Furthermore, the dire side effects of long-term medication use, poor patient compliance, and the prohibitive costs of treatment present significant challenges for many patients and their families (11-15).

For peer review only - http://bmjopen.bmj.com/site/about/guidelines.xhtml

Thus, it is imperative to offer an adjunctive therapeutic option from the perspective of psychotherapy to assist individuals with schizophrenia to live a fulfilling life alongside their condition.

Group arts therapies (GATs) represent a novel psychotherapeutic approach, utilizing the collective creation of art under professional guidance to support mental health (16-18). Employing mediums like painting, music, dance, and games, GATs focus on the composition and interaction of group members to boost mental well-being (19-21). Certain studies affirm the significant impact of GATs on the social functioning deficits (22), sense of pleasure (23), self-efficacy (24), and interpersonal relationships (25) of individuals with schizophrenia. The therapeutic benefits stem partly from the diverse artistic mediums that redirect patients' focus to the process of creation, helping to alleviate anxiety and stress, thus facilitating emotional expression and ameliorating the presentation of positive symptoms (26). Furthermore, by cultivating artistic skills, GATs improve self-confidence, emotional, cognitive, and social functioning (27). The interactive nature of GATs also promotes physiological, psychological, and social well-being through shared experiences and support within the therapy group (28). For individuals facing verbal communication challenges, GATs provide a platform for unencumbered emotional expression, potentially more effective than traditional verbal therapies (29). Listening to others share their artworks can help relieve negative symptoms (30,31). The advantage is especially evident in community settings, where collaborative art-making fosters meaningful interactions. The interaction can help these individuals overcome resistance and enhance their positive identity and personal recovery (32,33). Arts therapies in the community can also help in reducing the stigma of mental illness (34,35).

Despite these benefits, a systematic review of GATs' effectiveness in treating schizophrenia is lacking. This review aims to fill the gap by evaluating the evidence for GATs in clinical settings and offering a comparative analysis not present in existing literature.

Methods

Criteria for including studies in the review

Types of studies

All randomized controlled trials focused on patients diagnosed with schizophrenia will be included in the study. Scholarly journals published in Chinese, English, and Korean will constitute the primary publication types considered for inclusion. Duplicated publications, unpublished data, studies with inaccessible complete information, non-experimental reports, observational studies, and conference papers, literature reviews, case study reports, lectures, or critiques involving outcomes unrelated to GATs interventions will be excluded from this research.

Types of participants

Participants aged 18 to 70 years old, regardless of gender, ethnicity, nationality, educational level, or economic status, will be incorporated into the study. Diagnoses will be based on the Diagnostic and Statistical Manual of Mental Disorders (DSM)-III (36), DSM-III-R (37), DSM-IV (38), International Classification of Diseases (ICD)-10 criteria (39), or any other recognized standards. Individuals with significant organic injury, functional impairments, coexisting psychiatric conditions, or those who are unable to continue observation due to severe physical illness, as well as participants with incomplete clinical data, will be excluded from the study.

Types of interventions

Experiments comparing group-based arts therapies interventions with no treatment, standard pharmacotherapy, routine care, or any other form of active psychological counseling intervention will be included. Additionally, studies examining combined interventions, such as GATs in conjunction with standard pharmacotherapy, compared to solely utilizing an alternative modality, such as standard pharmacotherapy alone, also fall within the scope of this research.

Specifically, the British Association of Art Therapists (BAAT) definition of arts therapies was used as inclusion criteria for the intervention. BAAT proposes that arts therapies requires participants to use art materials for self-expression and reflection in

the presence of a trained art therapist. Therefore, the types of GATs that will be included in the study include painting, music, dance, yoga, drama, games, craft making, collage, photography, and film. Another necessary requirement is the involvement of qualified professionals such as art therapists or doctors during the research process. Additionally, settings in which interventions are delivered include inpatient, outpatient, and community settings. Furthermore, because in addition to necessary treatment settings such as hospitals and institutions, the community provides key rehabilitation intervention settings for people with schizophrenia that help improve social skills and quality of life, the settings in which interventions are implemented will include inpatient, outpatient, and community (41,42). Given the focus on arts therapies methods conducted within a group or community setting, individualized intervention measures will be excluded.

Types of outcomes

The primary outcomes will be the improvement of positive and negative symptoms in individuals with schizophrenia, to be measured using validated instruments such as the Positive and Negative Syndrome Scale (PANSS).

Secondary outcomes will encompass functional improvements associated with group arts therapies, including assessments of daily living capabilities, social functioning, emotional functioning, and cognitive functioning, as well as adverse events, compliance, costs, and cost-effectiveness. These outcomes will be evaluated using publicly recognized scales such as the Generic Quality of Life Inventory-74 (GQOLI-74), Personal and Social Performance scale (PSP), Nurses' Observation Scale for Inpatient Evaluation (NOSIE), and Tennessee Self-Concept Scale (TSCS), among others.

Electronic searches

Two researchers will conduct a search for all relevant literature published up to October 2023 in English, Chinese, and Korean. The literature will be sourced from databases such as PubMed, Web of Science, Cochrane Library, Embase, Wanfang Data, China National Knowledge Infrastructure (CNKI), RISS, Korean Citation Index (KCI), and

DBpia. The keyword combinations used will include: (a) group arts therapies, and (b) schizophrenia. The search strategy for PubMed is illustrated in Table 1 and appendix 1. Equivalent search strategies will be used in Chinese databases and Korean databases.

Table 1. Search strategies.

Number	Search terms
#1	Group Arts therapies
#2	Group Painting Therapy
#3	Group Music Therapy
#4	Group Dance Therapy
#5	Group Yoga Therapy
#6	Group Drama Therapy
#7	Group Handicrafts Therapy
#8	Group Collage Therapy
#9	Group Play Therapy
#10	Group Photography Therapy
#11	Group Movie Therapy
#12	#1 OR #2 OR #3 OR #4 OR #5 OR #6 OR #7 OR #8 OR #9 OR #10 OR #11
#13	Schizophrenia, schizophrenias
#14	Schizophrenic Disorders
#15	Schizophrenic Disorder
#16	Disorder, Schizophrenic
#17	Disorders, Schizophrenic
#18	#13 OR #14 OR #15 OR #16 OR #17
#20	#12 AND #18

For peer review only

Searching other resources

The reference lists of published relevant academic journals and review articles will also be manually searched to identify any missing literature. Furthermore, clinical guidelines pertaining to the relevant trials and articles of an ambiguous nature will be manually scrutinized to ensure no eligible studies are overlooked.

Data collection

Selection of studies

Two independent researchers will independently review the titles, abstracts, and full texts of the articles retrieved, compiling studies that meet the inclusion criteria. Any disagreements between researchers will be resolved through discussion to reach a consensus, and if necessary, a third independent reviewer will contribute to the final decision. The flowchart of the Preferred Reporting Items for Systematic Reviews and Meta-Analyses (PRISMA) delineates the selection process of the studies (Figure 1).

Data extraction and management

Two researchers will independently extract the following information from the included articles using a standardized form: (1) Basic information: first author's name, year of publication, region of publication, number of cases included, type of study design; (2) Clinical and pathological data: age, gender, duration of illness, length of hospital stay, inpatient environment; (3) Intervention measures: nature of the intervention, implementer, frequency of intervention per week, duration of the intervention; (4) Outcome measures; (5) Rating scales used. Both researchers will independently extract and code data according to this form. The results of the data extraction will be cross-checked. In case of discrepancies, a third researcher will be involved in the discussion and decision-making process.

Assessment of risk of bias

The risk of bias and methodological quality of all included randomized controlled trials will be assessed by two independent researchers using the Cochrane Risk of Bias 2 (RoB 2) tool (43). Each trial will be evaluated for bias according to the following domains: bias arising from the randomization process, bias due to deviations from the intended interventions, bias due to missing outcome data, bias in measurement of the outcome, and bias in selection of the reported result. Risks of bias will be classified as low, high, or unclear. In cases of disagreement, a third reviewer will be consulted, and consensus will be reached through discussion.

Data analysis

Measures of treatment effect

For peer review only - http://bmjopen.bmj.com/site/about/guidelines.xhtml

Review Manager (RevMan) 5.4 will be utilized to perform the meta-analysis. Continuous data will be expressed as mean difference (MD) and dichotomous data as risk ratio (RR), both with 95% confidence intervals (CI). When the same outcome is measured in various ways, the standardized mean difference (SMD) with a 95% CI will be chosen to represent continuous data.

Unit of analysis issues

The primary unit of analysis will be all individuals included in the control and experimental groups. Units of outcome measures from different studies will be converted to the International System of Units prior to analysis.

Dealing with missing data

Researchers will attempt to contact the authors included in the study via email to obtain missing or incomplete data. If missing data cannot be obtained, the study will be excluded.

Assessment of heterogeneity

Statistical heterogeneity among the included studies will be assessed using the I^2 statistic and the Chi^2 test. Studies will be considered heterogeneous when I2>50% or $p<0.1$.

Assessment of reporting biases

Reporting bias will be assessed following the CONSORT guidelines, and a funnel plot will be generated to evaluate potential reporting biases. A symmetric distribution of data within the funnel plot suggests the absence of publication bias. If the funnel plot is asymmetrical, efforts will be made to explore possible explanations beyond publication biases and language biases.

Data synthesis

Data synthesis will be conducted using RevMan 5.4. Results from two or more heterogeneous studies will be combined using a random effects model (I2>50% or

p<0.1). In the event that the meta-analysis is impeded by considerable clinical and methodological heterogeneity, a qualitative summary of the results will be provided.

Subgroup analysis and investigation of heterogeneity

In the presence of significant heterogeneity among the included studies, subgroup analyses will be conducted. Subgroups will be formed based on different populations (for example, male versus female), different types of GATs mediums (such as group painting, group music, or group play), and different art therapy settings (such as inpatient, outpatient, or community). The effect estimates for each subgroup will be calculated and compared inter-subgroup.

Sensitivity analysis

To ensure the robustness of the evidence, sensitivity analyses will be performed to assess the impact of studies with a high risk of bias and to explore possible sources of heterogeneity. Decisions to exclude lower-quality studies will be made by comparing outcomes and considering factors such as sample size, strength of evidence, and their influence on the pooled effective sample size.

Evidence quality evaluation

The quality of evidence will be further assessed using the Grading of Recommendations Assessment, Development and Evaluation (GRADE) approach, which categorizes the quality of evidence into four levels: very low, low, moderate, or high. In interpreting the findings, consideration will also be given to the quality of evidence, the context of the studies, potential benefits and harms, and patient values.

Patient and public involvement

Patients or the public were not involved in the study.

Ethics and dissemination

This systematic review does not require ethical approval, as all data utilized are anonymous and do not involve any private information. The outcomes of the research will offer evidence on the efficacy and safety of GATs as an adjunctive therapeutic

approach for schizophrenia and will be disseminated through peer-reviewed publications.

Discussion

While some explorations of GATs in the field of schizophrenia have been undertaken, evidence of its effectiveness in ameliorating positive, negative, and other related symptoms in patients remains limited. The inaugural MATISSE study on GATs indicated that GATs does not improve the overall functioning or mental health of individuals with schizophrenia (44), casting doubt on the overall efficacy of GATs. Since then, researchers have been seeking evidence to support the mechanistic changes induced by GATs. Evidence from randomized controlled trials of group painting therapy (45), group music therapy (46), group play therapy (47), and group dance therapy (48) indicate that group forms of treatment using different art media can effectively improve a variety of clinical outcomes in patients with schizophrenia. Additionally, a large-scale study is examining GATs' effectiveness in a diagnostically diverse patient population within mental health services (20), yielding promising initial findings. However, these investigations have not systematically examined the efficacy of different GAT modalities.

A review study on arts therapies for schizophrenia was conducted in 2005 (49). But due to the early start of the study, only two randomized controlled trials with insufficient participants were included, making the results less meaningful. A meta-analysis initiated in July 2021 intended to assess arts therapy effectiveness for schizophrenia failed to report outcomes. Uttley et al.'s systematic review highlighted potential cost-effectiveness of GATs for non-psychotic disorders, yet noted the evidence was too limited for a definitive comparison (50). Reviews on drama therapy (51), dance therapy (52), and music therapy (53) examined the benefits and harms of different types of arts therapies for patient with schizophrenia. However, similar to previous studies, there is only moderate to low-quality evidence that arts therapies can be used as a supplement to standard care to improve relevant clinical outcomes in schizophrenia. At the same time, these efforts are not conducted in groups or small groups. Whether subgroups

such as type, medium, duration, or frequency of GATs are related to treatment effectiveness remains unknown. The inclusion of large-scale and high-quality evidence and subgroup analyzes based on these variables may help address this issue.

This study aims to systematically assess the effectiveness of group-based artistic interventions in clinical treatment for schizophrenia, offering insights not provided by prior reviews. By examining a broader range of studies, including those published in English, Chinese, and Korean, this review seeks to enhance clinical evidence for healthcare providers and patients considering GATs as supplementary schizophrenia treatment.

Potential limitations of this study include possible language bias from excluding research in languages other than English, Chinese, and Korean, and publication bias due to the omission of conference papers, unpublished literature, and ongoing trials. Additionally, variability in GATs' types, mediums, durations, and frequencies may introduce heterogeneity. Nonetheless, this systematic approach still contributes to a broader understanding of GATs for schizophrenia.

Contributorship statement

AJZ wrote the draft of the manuscript, XXL and JW did independent reviews, RQL did research the reference. CHH, AJZ and RQL contributed to data organization, and JW and GHH critically reviewed the final draft of the manuscript. All authors contributed to the article and approved the submitted version.

Competing interests

The authors declared no conflict (competing) of interest.

Funding

This study is supported by the Research on Digital Art and Cultural Industry Development project of Guangzhou Wanqu Cooperative Institute of Design (9028).

Figure Legends

Figure1. PRISMA flow diagram of the study process.

Table Legends

Table 1. Search strategies.

Appendix Legends

Appendix 1. Full search strategies for all databases.

References

1. Correll, C. U., Solmi, M., Croatto, G., Schneider, L. K., Rohani-Montez, S. C., Fairley, L., ... & Tiihonen, J. (2022). Mortality in people with schizophrenia: a systematic review and meta-analysis of relative risk and aggravating or attenuating factors. World Psychiatry, 21(2), 248-271. doi: 10.1002/wps.20994

2. Crespo-Facorro, B., Such, P., Nylander, A. G., Madera, J., Resemann, H. K., Worthington, E., ... & Newton, R. (2021). The burden of disease in early schizophrenia–a systematic literature review. Current Medical Research and Opinion, 37(1), 109-121. doi: 10.1080/03007995.2020.1841618

3. Andreasen, N. (1995). Symptoms, signs, and diagnosis of schizophrenia. The Lancet, 346(8973), 477-481. doi: 10.1016/S0140-6736(95)91325-4

4. Temmingh, H., & Stein, D. J. (2015). Anxiety in patients with schizophrenia: Epidemiology and management. CNS drugs, 29, 819-832. doi: 10.1007/s40263-015-0282-7

5. Marder, S. R., & Galderisi, S. (2017). The current conceptualization of negative symptoms in schizophrenia. World Psychiatry, 16(1), 14-24. doi: 10.1002/wps.20385

6. Buchanan, R. W. (2007). Persistent negative symptoms in schizophrenia: an overview. Schizophrenia bulletin, 33(4), 1013-1022. doi: 10.1093/schbul/sbl057

7. Milev, P., Ho, B. C., Arndt, S., & Andreasen, N. C. (2005). Predictive values of neurocognition and negative symptoms on functional outcome in schizophrenia: a longitudinal first-episode study with 7-year follow-up. American Journal of Psychiatry, 162(3), 495-506. doi: 10.1176/appi.ajp.162.3.495

8. Leucht, S., Tardy, M., Komossa, K., Heres, S., Kissling, W., Salanti, G., & Davis, J. M. (2012). Antipsychotic drugs versus placebo for relapse prevention in schizophrenia: a systematic review and meta-analysis. The Lancet, 379(9831), 2063-2071. doi: 10.1016/S0140-6736（12）60239-6

9. Kane, J. M., & Correll, C. U. (2010). Past and present progress in the pharmacologic treatment of schizophrenia. The Journal of clinical psychiatry, 71(9), 13909. doi：10.4088/JCP.10r06264yel

10. Kirkpatrick, B., Fenton, W. S., Carpenter Jr, W. T., & Marder, S. R. (2006). The NIMH-MATRICS consensus statement on negative symptoms. Schizophrenia bulletin, 32(2), 214-219. doi: 10.1093/schbul/sbj053

11. De las Cuevas, C., Sanz, E. J., & de Leon, J. (2023). Adverse drug reactions and their fatal outcomes in clozapine patients in VigiBase: Comparing the top four reporting countries (US, UK, Canada and Australia). Schizophrenia Research. doi: 10.1016/j.schres.2023.05.004

12. Papola, D., Ostuzzi, G., Gastaldon, C., Morgano, G. P., Dragioti, E., Carvalho, A. F., ... & Barbui, C. (2019). Antipsychotic use and risk of life - threatening medical events: umbrella review of observational studies. Acta Psychiatrica Scandinavica, 140(3), 227-243. doi: 10.1111/acps.13066

13. Lindström, E., & Bingefors, K. (2000). Patient compliance with drug therapy in schizophrenia: economic and clinical issues. Pharmacoeconomics, 18, 105-124. doi: 10.2165/00019053-200018020-00002

14. Peng, M. M., Xing, J., Tang, X., Wu, Q., Wei, D., & Ran, M. S. (2022). Disease-related risk factors for caregiver burden among family caregivers of persons with schizophrenia: a systematic review and meta-analysis. International Journal of Environmental Research and Public Health, 19(3), 1862. doi: 10.3390/ijerph19031862

15. Jin, H., & Mosweu, I. (2017). The societal cost of schizophrenia: a systematic review. Pharmacoeconomics, 35, 25-42. doi: 10.1007/s40273-016-0444-6

16. Moon, B. L. (2016). Art-based group therapy: Theory and practice. Charles C Thomas Publisher.

17. Liebmann, M. (2004). Art therapy for groups: A handbook of themes and exercises. Psychology Press.

18. Hui, H. (2023). Intervention Study of Expressive Art Group Therapy on Children with Emotional Distress. Advances in Psychology, 13(7), 2757-2765. https://doi.org/10.12677/AP.2023.137340

19. American Group Psychotherapy Association Science to Service Task Force. Practice guidelines for group psychotherapy. American Group Psychotherapy Association; 2007.

20. Carr, C. E., Medlicott, E., Hooper, R., Feng, Y., Mihaylova, B., & Priebe, S. (2023). Effectiveness of group arts therapies (art therapy, dance movement therapy and music therapy) compared to group counselling for diagnostically heterogeneous psychiatric community patients: study protocol for a randomised controlled trial in mental health services (the ERA study). Trials, 24(1), 557. https://doi.org/10.1186/s13063-023-07232-0

21. Karkou V, Sanderson P. (2005). Arts therapies: a research-based map of the field. London: Elsevier Churchill Livingstone.

22. Orfanos, S., Banks, C., & Priebe, S. (2015). Are group psychotherapeutic treatments effective for patients with schizophrenia? A systematic review and meta-analysis. Psychotherapy and psychosomatics, 84(4), 241-249. https://doi.org/10.1159/000377705

23. Cho, J. M., & Lee, K. (2018). Effects of motivation interviewing using a group art therapy program on negative symptoms of schizophrenia. Archives of psychiatric nursing, 32(6), 878-884. https://doi.org/10.1016/j.apnu.2018.07.002

24. Tong, J., Yu, W., Fan, X., Sun, X., Zhang, J., Zhang, J., & Zhang, T. (2021). Impact of group art therapy using traditional Chinese materials on self-efficacy and social function for individuals diagnosed with schizophrenia. Frontiers in Psychology, 11, 571124. https://doi.org/10.3389/fpsyg.2020.571124

25. Green, B. L., Wehling, C., & Taisky, G. J. (1987). Group art therapy as an adjunct to treatment for chronic outpatients. Psychiatric Services, 38(9), 988-991. https://doi.org/10.1176/ps.38.9.988

26. Waller, D. (2014). Group interactive art therapy: Its use in training and treatment. Routledge.

27. Chandraiah, S., Ainlay Anand, S., & Avent, L. C. (2012). Efficacy of group art therapy on depressive symptoms in adult heterogeneous psychiatric outpatients. Art Therapy, 29(2), 80-86. doi: 10.1080/07421656.2012.683739

28. Waller, D., & Gilroy, A. (2000). Art Therapy: A handbook of new theories and practice. Buckingham: Open University Press.

29. Gabel, A., & Robb, M. (2017). (Re) considering psychological constructs: A thematic synthesis defining five therapeutic factors in group art therapy. The arts in psychotherapy, 55, 126-135. doi: 10.1016/j.aip.2017.05.005

30. Jewell, M., & Camden-Pratt, C. (2023). Creating 'art-alongside'in Peer Art Therapy (PATh) groups: nurturing connection and trust, and responding to power

dynamics. International Journal of Art Therapy, 28:1-2, 38-50. doi: 10.1080/17454832.2023.2175002

31. Springham, N., & Camic, P. M. (2017). Observing mentalizing art therapy groups for people diagnosed with borderline personality disorder. International Journal of Art Therapy, 22(3), 138-152. https://doi.org/10.1080/17454832.2017.1288753

32. Rainbow T. H. Ho, Jordan S. Potash, Andy H. Y. Ho, Vania F. L. Ho & Eric Y. H. Chen. (2017). Reducing mental illness stigma and fostering empathic citizenship: Community arts collaborative approach, Social Work in Mental Health, 15:4, 469-485. DOI: 10.1080/15332985.2016.1236767

33. Kapitan, L., Litell, M., & Torres, A. (2011). Creative art therapy in a community's participatory research and social transformation. Art Therapy, 28(2), 64-73. DOI: 10.1080/07421656.2011.578238

34. Oren, R., Orkibi, H., Elefant, C., & Salomon-Gimmon, M. (2019). Arts-based psychiatric rehabilitation programs in the community: Perceptions of healthcare professionals. Psychiatric Rehabilitation Journal, 42(1), 41–47. https://doi.org/10.1037/prj0000325

35. Armijo, J., Méndez, E., Morales, R., Schilling, S., Castro, A., Alvarado, R., & Rojas, G. (2013). Efficacy of community treatments for schizophrenia and other psychotic disorders: a literature review. Frontiers in psychiatry, 4, 116. https://doi.org/10.3389/fpsyt.2013.00116

36. Spitzer, R. L., Williams, J. B., & Skodol, A. E. (1980). DSM-III: the major achievements and an overview. The American Journal of Psychiatry, 137(2), 151-164. https://doi.org/10.1176/ajp.137.2.151

37. Widiger, T. A., Frances, A., Spitzer, R. L., & Williams, J. B. (1988). The DSM-III-R personality disorders: an overview. The American Journal of Psychiatry, 145(7), 786-795. https://doi.org/10.1176/ajp.145.7.786

38. Frances, A., First, M. B., & Pincus, H. A. (1995). DSM-IV guidebook. American Psychiatric Association.

39. World Health Organization. (1992). The ICD-10 classification of mental and behavioural disorders: clinical descriptions and diagnostic guidelines (Vol. 1). World Health Organization.

40. Edwards D. (2004). Art therapy. Sage.

41. Gowda, G. S., & Isaac, M. K. (2022). Models of care of schizophrenia in the community—an international perspective. Current psychiatry reports, 24(3), 195-202. https://doi.org/10.1007/s11920-022-01329-0

42. Lim, C., Barrio, C., Hernandez, M., Barragán, A., Yamada, A.-M., & Brekke, J. S. (2016). Remission of symptoms in community-based psychosocial rehabilitation services for individuals with schizophrenia. Psychiatric Rehabilitation Journal, 39(1), 42–46. https://doi.org/10.1037/prj0000154

43. Higgins, J. P., Sterne, J. A., Savovic, J., Page, M. J., Hróbjartsson, A., Boutron, I., ... & Eldridge, S. (2016). A revised tool for assessing risk of bias in randomized trials. Cochrane database of systematic reviews, 10 (Suppl 1), 29-31. dx.doi.org/10.1002/14651858.CD201601

44. Crawford, M. J., Killaspy, H., Kalaitzaki, E., Barrett, B., Byford, S., Patterson, S., ... & Waller, D. (2010). The MATISSE study: a randomised trial of group art therapy for people with schizophrenia. BMC psychiatry, 10(1), 1-9. https://doi.org/10.1186/1471-244X-10-65

45. Cho, J. M., & Lee, K. (2018). Effects of motivation interviewing using a group art therapy program on negative symptoms of schizophrenia. Archives of psychiatric nursing, 32(6), 878-884. https://doi.org/10.1016/j.apnu.2018.07.002

46. Peng, S. M., Koo, M., & Kuo, J. C. (2010). Effect of group music activity as an adjunctive therapy on psychotic symptoms in patients with acute schizophrenia. Archives of psychiatric nursing, 24(6), 429-434. https://doi.org/10.1016/j.apnu.2010.04.001

47. Torres, A., Mendez, L. P., Merino, H., & Moran, E. A. (2002). Rehab rounds: Improving social functioning in schizophrenia by playing the train game. Psychiatric Services, 53(7), 799-801. https://doi.org/10.1176/appi.ps.53.7.799

48. Bryl, K., Bradt, J., Cechnicki, A., Fisher, K., Sossin, K. M., & Goodill, S. (2022). The role of dance/movement therapy in the treatment of negative symptoms in schizophrenia: a mixed methods pilot study. Journal of Mental Health, 31(5), 613-623. https://doi.org/10.1080/09638237.2020.1757051

49. Ruddy, R., & Milnes, D. (2005). Art therapy for schizophrenia or schizophrenia ‐ like illnesses. Cochrane Database of Systematic Reviews, (4). https://doi.org/10.1002/14651858.CD003728.pub2

50. Uttley, L., Stevenson, M., Scope, A., Rawdin, A., & Sutton, A. (2015). The clinical and cost effectiveness of group art therapy for people with non-psychotic mental health disorders: a systematic review and cost-effectiveness analysis. BMC psychiatry, 15(1), 1-13. https://doi.org/10.1186/s12888-015-0528-4

51. Ruddy, R., & Dent ‐ Brown, K. (2007). Drama therapy for schizophrenia or schizophrenia ‐ like illnesses. Cochrane Database of Systematic Reviews, (1). https://doi.org/10.1002/14651858.CD005378.pub2

462 52. Ren, J., & Xia, J. (2013). Dance therapy for schizophrenia. Cochrane Database of Systematic Reviews, (10). https://doi.org/10.1002/14651858.CD006868.pub3

464 53. Geretsegger, M., Mössler, K. A., Bieleninik, Ł., Chen, X. J., Heldal, T. O., & Gold, C. (2017). Music therapy for people with schizophrenia and schizophrenia - like disorders. Cochrane Database of Systematic Reviews, (5). https://doi.org/10.1002/14651858.CD004025.pub4

For peer review only

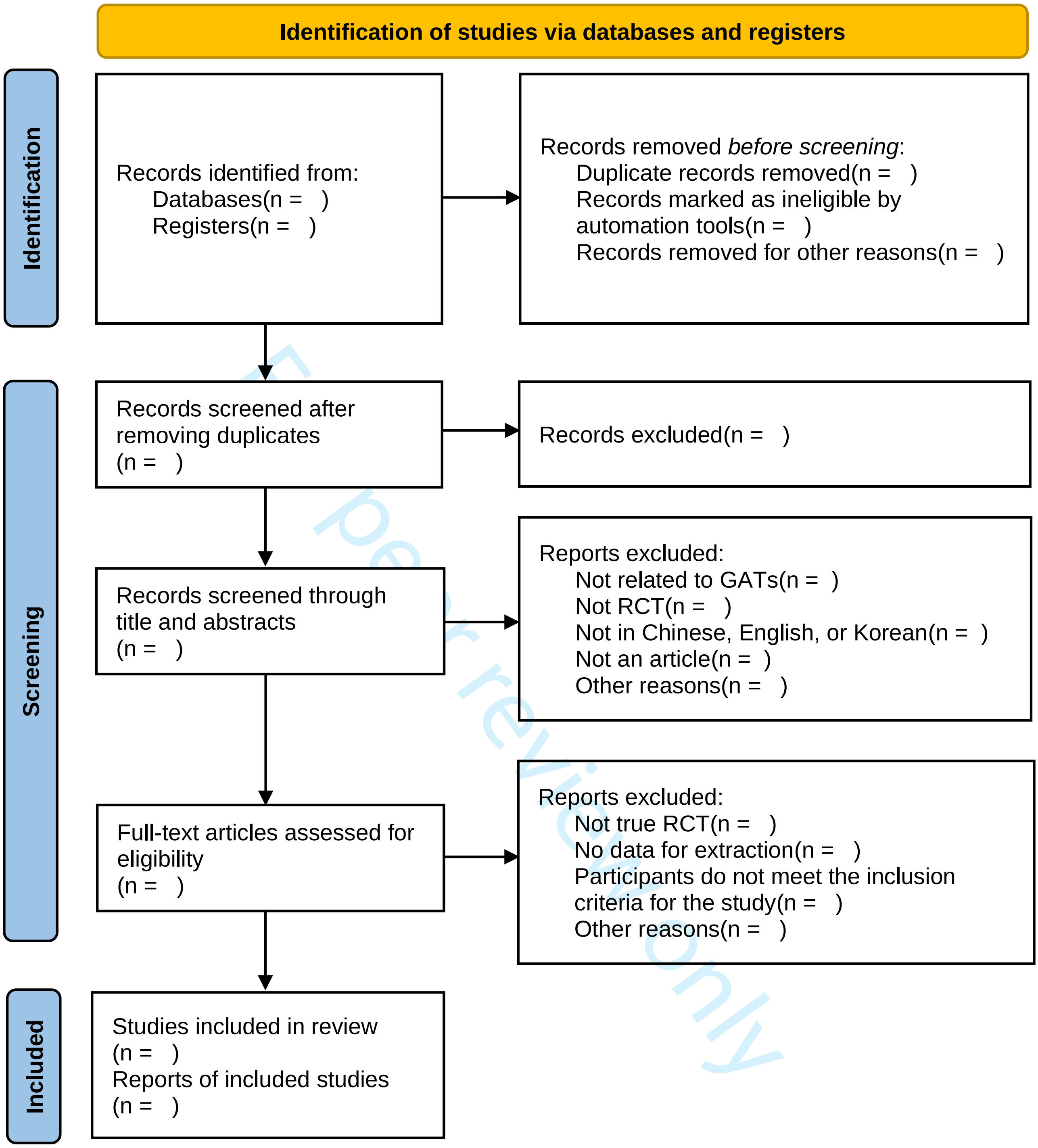

Figure 1. PRISMA flow diagram of the study process. PRISMA, Preferred Reporting Items for Systematic review and Meta-Analysis; RCT, randomized controlled trial; GATs, group arts therapies.

Group Arts Therapies for Patients with Schizophrenia: A Protocol of Systematic Review and Meta-analysis

Appendix 1. Full search strategies for all databases.

Database	Search strategy
PubMed	((((((((((((((group arts therapies[Title/Abstract]) OR (group painting therapy[Title/Abstract])) OR (group music therapy[Title/Abstract])) OR (group dance therapy[Title/Abstract])) OR (group yoga therapy[Title/Abstract])) OR (group drama therapy[Title/Abstract])) OR (group handicrafts therapy[Title/Abstract])) OR (group collage therapy[Title/Abstract])) OR (group play therapy[Title/Abstract])) OR (group photography therapy[Title/Abstract])) OR (group movie therapy[Title/Abstract])) AND (Schizophrenia, schizophrenias[Title/Abstract])) OR (Schizophrenic Disorders[Title/Abstract])) OR (Schizophrenic Disorder[Title/Abstract])) OR (Disorder, Schizophrenic[Title/Abstract])) OR (Disorders, Schizophrenic[Title/Abstract])
Web of Science	(ALL=(Group Arts therapies)) OR ALL=(Group Painting Therapy)) OR ALL=(Group Music Therapy)) OR ALL=(Group Dance Therapy)) OR ALL=(Group Yoga Therapy)) OR ALL=(Group Drama Therapy)) OR ALL=(Group Handicrafts Therapy)) OR ALL=(Group Collage Therapy)) OR ALL=(Group Play Therapy)) OR ALL=(Group Photography Therapy)) OR ALL=(Group Movie Therapy)) AND ALL=(Schizophrenia, schizophrenias)) OR ALL=(Schizophrenic Disorders)) OR ALL=(Schizophrenic Disorder)) OR ALL=(Disorder, Schizophrenic)) OR ALL=(Disorders, Schizophrenic)
Cochrane Library	Number — Search terms<br>#1 Group Arts therapies<br>#2 Group Painting Therapy<br>#3 Group Music Therapy<br>#4 Group Dance Therapy<br>#5 Group Yoga Therapy<br>#6 Group Drama Therapy<br>#7 Group Handicrafts Therapy<br>#8 Group Collage Therapy<br>#9 Group Play Therapy<br>#10 Group Photography Therapy<br>#11 Group Movie Therapy<br>#12 #1 OR #2 OR #3 OR #4 OR #5 OR #6 OR #7 OR #8 OR #9 OR #10 OR #11<br>#13 Schizophrenia, schizophrenias<br>#14 Schizophrenic Disorders<br>#15 Schizophrenic Disorder<br>#16 Disorder, Schizophrenic<br>#17 Disorders, Schizophrenic<br>#18 #13 OR #14 OR #15 OR #16 OR #17<br>#20 #12 AND #18
Embase	('group arts therapies' OR (('group'/exp OR group) AND arts AND therapies) OR 'group painting therapy' OR (('group'/exp OR group) AND

For peer review

	('painting'/exp OR painting) AND ('therapy'/exp OR therapy)) OR 'group music therapy' OR (('group'/exp OR group) AND ('music'/exp OR music) AND ('therapy'/exp OR therapy)) OR 'group dance therapy' OR (('group'/exp OR group) AND ('dance'/exp OR dance) AND ('therapy'/exp OR therapy)) OR 'group yoga therapy' OR (('group'/exp OR group) AND ('yoga'/exp OR yoga) AND ('therapy'/exp OR therapy)) OR 'group drama therapy' OR (('group'/exp OR group) AND ('drama'/exp OR drama) AND ('therapy'/exp OR therapy)) OR 'group handicrafts therapy' OR (('group'/exp OR group) AND handicrafts AND ('therapy'/exp OR therapy)) OR 'group collage therapy' OR (('group'/exp OR group) AND collage AND ('therapy'/exp OR therapy)) OR 'group play therapy' OR (('group'/exp OR group) AND ('play'/exp OR play) AND ('therapy'/exp OR therapy)) OR 'group photography therapy' OR (('group'/exp OR group) AND ('photography'/exp OR photography) AND ('therapy'/exp OR therapy)) OR 'group movie therapy' OR (('group'/exp OR group) AND ('movie'/exp OR movie) AND ('therapy'/exp OR therapy))) AND ('schizophrenia, schizophrenias' OR (('schizophrenia,'/exp OR schizophrenia,) AND schizophrenias) OR 'schizophrenic disorders' OR (('schizophrenic'/exp OR schizophrenic) AND ('disorders'/exp OR disorders)) OR 'schizophrenic disorder' OR (('schizophrenic'/exp OR schizophrenic) AND ('disorder'/exp OR disorder)) OR 'disorder, schizophrenic' OR (('disorder,'/exp OR disorder,) AND ('schizophrenic'/exp OR schizophrenic)) OR 'disorders, schizophrenic' OR (('disorders,'/exp OR disorders,) AND ('schizophrenic'/exp OR schizophrenic)))
China National Knowledge Infrastructure (CNKI)	（团体艺术治疗 OR 团体绘画疗法 OR 团体音乐疗法 OR 团体舞蹈疗法 OR 团体瑜伽疗法 OR 团体戏剧疗法 OR 团体手工艺疗法 OR 团体拼贴画疗法 OR 团体游戏疗法 OR 团体摄影疗法 OR 团体电影疗法） AND （精神分裂症 OR 精神分裂）
Wanfang Data	（团体艺术治疗 或 团体绘画疗法 或 团体音乐疗法 或 团体舞蹈疗法 或 团体瑜伽疗法 或 团体戏剧疗法 或 团体手工艺疗法 或 团体拼贴画疗法 或 团体游戏疗法 或 团体摄影疗法 或 团体电影疗法） 与 （精神分裂症 或 精神分裂）
RISS	(((((전체=(집단 예술 치료) OR 전체=(집단 미술 치료) OR 전체=(집단 음악 치료) OR 전체=(집단 무용 치료) OR 전체=(집단 요가 치료) OR 전체=(집단 드라마 치료) OR 전체=(집단 수공예 치료) OR 전체=(집단 콜라주 치료) OR 전체=(집단 놀이 치료) OR 전체=(집단 사진 치료) OR 전체=(집단 영화 치료)) AND 전체=(조현병)) OR 전체=(정신분열증))
Korean Citation Index (KCI)	(((((전체=(집단 예술 치료) OR 전체=(집단 미술 치료) OR 전체=(집단 음악 치료) OR 전체=(집단 무용 치료) OR 전체=(집단 요가 치료) OR 전체=(집단 드라마 치료) OR 전체=(집단 수공예 치료) OR 전체=(집단 콜라주 치료) OR 전체=(집단 놀이 치료) OR 전체=(집단 사진 치료) OR 전체=(집단 영화 치료)) AND 전체=(조현병)) OR 전체=(정신분열증))

For peer review only

DBpia	(((((전체=(집단 예술 치료) OR 전체=(집단 미술 치료) OR 전체=(집단 음악 치료) OR 전체=(집단 무용 치료) OR 전체=(집단 요가 치료) OR 전체=(집단 드라마 치료) OR 전체=(집단 수공예 치료) OR 전체=(집단 콜라주 치료) OR 전체=(집단 놀이 치료) OR 전체=(집단 사진 치료) OR 전체=(집단 영화 치료)) AND 전체=(조현병)) OR 전체=(정신분열증))

For peer review only

PRISMA-P (Preferred Reporting Items for Systematic review and Meta-Analysis Protocols) 2015 checklist: recommended items to address in a systematic review protocol*

	Section and topic	Item No	Checklist item
	**ADMINISTRATIVE INFORMATION**		
1-2	Title:		
	Identification	1a	Identify the report as a protocol of a systematic review
	Update	1b	If the protocol is for an update of a previous systematic review, identify as such
29	Registration	2	If registered, provide the name of the registry (such as PROSPERO) and registration number
Anonymization of submission documents	Authors:		
	Contact	3a	Provide name, institutional affiliation, e-mail address of all protocol authors; provide physical mailing address of corresponding author
	Contributions	3b	Describe contributions of protocol authors and identify the guarantor of the review
/	Amendments	4	If the protocol represents an amendment of a previously completed or published protocol, identify as such and list changes; otherwise, state plan for documenting important protocol amendments
288-289	Support:		
	Sources	5a	Indicate sources of financial or other support for the review
	Sponsor	5b	Provide name for the review funder and/or sponsor
	Role of sponsor or funder	5c	Describe roles of funder(s), sponsor(s), and/or institution(s), if any, in developing the protocol
	**INTRODUCTION**		
42-81	Rationale	6	Describe the rationale for the review in the context of what is already known
82-85	Objectives	7	Provide an explicit statement of the question(s) the review will address with reference to participants, interventions, comparators, and outcomes (PICO)
	**METHODS**		
88-152	Eligibility criteria	8	Specify the study characteristics (such as PICO, study design, setting, time frame) and report characteristics (such as years considered, language, publication status) to be used as criteria for eligibility for the review
140-152	Information sources	9	Describe all intended information sources (such as electronic databases, contact with study authors, trial registers or other grey literature sources) with planned dates of coverage
table 1 appendix 1	Search strategy	10	Present draft of search strategy to be used for at least one electronic database, including planned limits, such that it could be repeated
	Study records:		
165-174	Data management	11a	Describe the mechanism(s) that will be used to manage records and data throughout the review

For peer review only

155-163	Selection process	11b	State the process that will be used for selecting studies (such as two independent reviewers) through each phase of the review (that is, screening, eligibility and inclusion in meta-analysis)
165-174	Data collection process	11c	Describe planned method of extracting data from reports (such as piloting forms, done independently, in duplicate), any processes for obtaining and confirming data from investigators
165-174	Data items	12	List and define all variables for which data will be sought (such as PICO items, funding sources), any pre-planned data assumptions and simplifications
128-138	Outcomes and prioritization	13	List and define all outcomes for which data will be sought, including prioritization of main and additional outcomes, with rationale
176-183	Risk of bias in individual studies	14	Describe anticipated methods for assessing risk of bias of individual studies, including whether this will be done at the outcome or study level, or both; state how this information will be used in data synthesis
210-213	Data synthesis	15a	Describe criteria under which study data will be quantitatively synthesised
		15b	If data are appropriate for quantitative synthesis, describe planned summary measures, methods of handling data and methods of combining data from studies, including any planned exploration of consistency (such as I^2, Kendall's τ)
		15c	Describe any proposed additional analyses (such as sensitivity or subgroup analyses, meta-regression)
		15d	If quantitative synthesis is not appropriate, describe the type of summary planned
204-208 and 228-226	Meta-bias(es)	16	Specify any planned assessment of meta-bias(es) (such as publication bias across studies, selective reporting within studies)
228-232	Confidence in cumulative evidence	17	Describe how the strength of the body of evidence will be assessed (such as GRADE)

*** It is strongly recommended that this checklist be read in conjunction with the PRISMA-P Explanation and Elaboration (cite when available) for important clarification on the items. Amendments to a review protocol should be tracked and dated. The copyright for PRISMA-P (including checklist) is held by the PRISMA-P Group and is distributed under a Creative Commons Attribution Licence 4.0.**

From: Shamseer L, Moher D, Clarke M, Ghersi D, Liberati A, Petticrew M, Shekelle P, Stewart L, PRISMA-P Group. Preferred reporting items for systematic review and meta-analysis protocols (PRISMA-P) 2015: elaboration and explanation. BMJ. 2015 Jan 2;349(jan02 1):g7647.

For peer review only

Group Arts Therapies for Patients with Schizophrenia: A Protocol of Systematic Review and Meta-analysis

Journal:	*BMJ Open*
Manuscript ID	bmjopen-2023-082076.R2
Article Type:	Protocol
Date Submitted by the Author:	01-Apr-2024
Complete List of Authors:	Zhang, Aijia; Macau University of Science and Technology, Faculty of Humanities and Arts<br>Luo, Xuexing; Macau University of Science and Technology<br>Lin, Runqing; Macau University of Science and Technology<br>He, Caihong; Guangzhou Wanqu Cooperative Institute of Design<br>Wang, Jue; Macau University of Science and Technology, ; Macau University of Science and Technology<br>Huang, Guanghui; Macau University of Science and Technology; Zhuhai MUST Science and Technology Research Institute
**Primary Subject Heading**:	Mental health
Secondary Subject Heading:	Qualitative research, Neurology, Patient-centred medicine
Keywords:	Physiology < NATURAL SCIENCE DISCIPLINES, Meta-Analysis, Schizophrenia & psychotic disorders < PSYCHIATRY

SCHOLARONE™
Manuscripts

Group Arts Therapies for Patients with Schizophrenia: A Protocol of Systematic Review and Meta-analysis

Abstract

Introduction: Schizophrenia, a chronic mental problem, significantly impacts cognition, emotion, and social functioning. Conventional pharmacotherapy faces challenges including numerous side effects, low adherence to medication, and substantial costs. In this context, Group Arts Therapies (GATs) emerge as a promising complementary approach for symptom alleviation in schizophrenia patients. Nonetheless, the effectiveness and safety of GATs are yet to be firmly established. This study aims to systematically assess the therapeutic impact of all group-based artistic interventions as complementary treatments for schizophrenia, focusing on their potential benefits.

Methods and analysis: This study will search four English-language databases (PubMed, Web of Science, Cochrane Library, Embase), two Chinese databases (Wanfang Data, China National Knowledge Infrastructure), and three Korean databases (RISS, Korean Citation Index, DBpia) from their inception until October 2023. It will include all randomized controlled trials (RCTs) that compare Group Arts Therapies (GATs) for schizophrenia with standard rehabilitation methods. The primary outcome is the improvement in patients' positive and negative symptoms. Methodologies such as bias risk assessment, data synthesis, sensitivity analysis, and subgroup analysis will be implemented using Review Manager 5.4. Study results with high heterogeneity will be merged using a random-effects model ($I^2 > 50\%$ or $p < 0.1$). In cases where meta-analysis is not viable due to significant clinical and methodological heterogeneity, a qualitative summary of the findings will be provided.

Ethics and dissemination: The data utilized in this systematic review are anonymized, devoid of any private information, eliminating the requirement for ethical approval. Dissemination of the research findings will be conducted via peer-reviewed publications.

PROSPERO registration number: CRD42023471583.

For peer review only

Strengths and limitations of this study

- This systematic review intends to comprehensively appraise the efficacy of group-based therapeutic approaches in art for schizophrenia, incorporating diverse modalities such as painting, music, dance, yoga, drama, handicrafts, collage, play, photography, and movie.
- The study displays relatively minor language bias, as research published in Chinese and Korean has been included alongside English-language studies.
- Potential publication bias may exist due to the exclusion of conference papers, unpublished documents, and ongoing clinical trials from the included literature.
- Significant heterogeneity might be present owing to variations in the type of GAT, as well as differences in the duration and frequency of the treatment.

Introduction

Individuals with schizophrenia endure significant physiological and psychological stress. A recent epidemiological survey shows that these individuals face a higher risk of suicide and unemployment compared to the general population (1,2). This increased risk is attributed to the presence of positive symptoms such as hallucinations, delusions, and disordered thinking (3). Furthermore, physiological distress intensifies feelings of anxiety and depression, potentially leading to dire decisions (4). The condition is also characterized by negative symptoms, including cognitive impairments, emotional flatness, social withdrawal, and diminished motivation, which severely limit the ability to participate in social life. These symptoms are the primary causes for their long-term incidence rate and may struggle to undertake activities of daily living (5-7).

At present, pharmacotherapy serves as the principal treatment approach for schizophrenia, showing relative efficacy in mitigating positive symptoms and preventing relapse (8,9). However, studies suggest that medication alone has limited effectiveness in reducing negative symptoms (10). Furthermore, the dire side effects of long-term medication use, poor patient compliance, and the prohibitive costs of treatment present significant challenges for many patients and their families (11-15).

Thus, it is imperative to offer an adjunctive therapeutic option from the perspective of psychotherapy to assist individuals with schizophrenia to live a fulfilling life alongside their condition.

Group arts therapies (GATs) represent a novel psychotherapeutic approach, utilizing the collective creation of art under professional guidance to support mental health (16-18). Employing mediums like painting, music, dance, and games, GATs focus on the composition and interaction of group members to boost mental well-being (19-21). Certain studies affirm the significant impact of GATs on the social functioning deficits (22), sense of pleasure (23), self-efficacy (24), and interpersonal relationships (25) of individuals with schizophrenia. The therapeutic benefits stem partly from the diverse artistic mediums that redirect patients' focus to the process of creation, helping to alleviate anxiety and stress, thus facilitating emotional expression and ameliorating the presentation of positive symptoms (26). Furthermore, by cultivating artistic skills, GATs improve self-confidence, emotional, cognitive, and social functioning (27). The interactive nature of GATs also promotes physiological, psychological, and social well-being through shared experiences and support within the therapy group (28). For individuals facing verbal communication challenges, GATs provide a platform for unencumbered emotional expression, potentially more effective than traditional verbal therapies (29). Listening to others share their artworks can help relieve negative symptoms (30,31). The advantage is especially evident in community settings, where collaborative art-making fosters meaningful interactions. The interaction can help these individuals overcome resistance and enhance their positive identity and personal recovery (32,33). Arts therapies in the community can also help in reducing the stigma of mental illness (34,35).

Despite these benefits, a systematic review of GATs' effectiveness in treating schizophrenia is lacking. This review aims to fill the gap by evaluating the evidence for GATs in clinical settings and offering a comparative analysis not present in existing literature.

Methods

Criteria for including studies in the review

Types of studies

All randomized controlled trials focused on patients diagnosed with schizophrenia will be included in the study. Scholarly journals published in Chinese, English, and Korean will constitute the primary publication types considered for inclusion. Duplicated publications, unpublished data, studies with inaccessible complete information, non-experimental reports, observational studies, and conference papers, literature reviews, case study reports, lectures, or critiques involving outcomes unrelated to GATs interventions will be excluded from this research.

Types of participants

Participants aged 18 to 70 years old, regardless of gender, ethnicity, nationality, educational level, or economic status, will be incorporated into the study. Diagnoses will be based on the Diagnostic and Statistical Manual of Mental Disorders (DSM)-III (36), DSM-III-R (37), DSM-IV (38), International Classification of Diseases (ICD)-10 criteria (39), or any other recognized standards. Individuals with significant organic injury, functional impairments, coexisting psychiatric conditions, or those who are unable to continue observation due to severe physical illness, as well as participants with incomplete clinical data, will be excluded from the study.

Types of interventions

Experiments comparing group-based arts therapies interventions with no treatment, standard pharmacotherapy, routine care, or any other form of active psychological counseling intervention will be included. Additionally, studies examining combined interventions, such as GATs in conjunction with standard pharmacotherapy, compared to solely utilizing an alternative modality, such as standard pharmacotherapy alone, also fall within the scope of this research.

Specifically, the British Association of Art Therapists (BAAT) definition of arts therapies was used as inclusion criteria for the intervention. BAAT proposes that arts therapies require participants to use art materials for self-expression and reflection in

the presence of a trained art therapist (40). Therefore, the types of GATs that will be included in the study include painting, music, dance, yoga, drama, games, craft making, collage, photography, and film. Another necessary requirement is the involvement of qualified professionals such as art therapists or doctors during the research process. Additionally, settings in which interventions are delivered include inpatient, outpatient, and community settings. Furthermore, because in addition to necessary treatment settings such as hospitals and institutions, the community provides key rehabilitation intervention settings for people with schizophrenia that help improve social skills and quality of life, the settings in which interventions are implemented will include inpatient, outpatient, and community (41,42). Given the focus on arts therapies methods conducted within a group or community setting, individualized intervention measures will be excluded.

Types of outcomes

The primary outcomes will be the improvement of positive and negative symptoms in individuals with schizophrenia, to be measured using validated instruments such as the Positive and Negative Syndrome Scale (PANSS).

Secondary outcomes will encompass functional improvements associated with group arts therapies, including assessments of daily living capabilities, social functioning, emotional functioning, and cognitive functioning, as well as adverse events, compliance, costs, and cost-effectiveness. These outcomes will be evaluated using publicly recognized scales such as the Generic Quality of Life Inventory-74 (GQOLI-74), Personal and Social Performance scale (PSP), Nurses' Observation Scale for Inpatient Evaluation (NOSIE), and Tennessee Self-Concept Scale (TSCS), among others.

Electronic searches

Two researchers will conduct a search for all relevant literature published up to October 2023 in English, Chinese, and Korean. The literature will be sourced from databases such as PubMed, Web of Science, Cochrane Library, Embase, Wanfang Data, China National Knowledge Infrastructure (CNKI), RISS, Korean Citation Index (KCI), and

DBpia. The keyword combinations used will include: (a) group arts therapies, and (b) schizophrenia. The search strategy for PubMed is illustrated in Table 1 and appendix 1. Equivalent search strategies will be used in Chinese databases and Korean databases.

Table 1. Search strategies.

Number	Search terms
#1	Group Arts therapies
#2	Group Painting Therapy
#3	Group Music Therapy
#4	Group Dance Therapy
#5	Group Yoga Therapy
#6	Group Drama Therapy
#7	Group Handicrafts Therapy
#8	Group Collage Therapy
#9	Group Play Therapy
#10	Group Photography Therapy
#11	Group Movie Therapy
#12	#1 OR #2 OR #3 OR #4 OR #5 OR #6 OR #7 OR #8 OR #9 OR #10 OR #11
#13	Schizophrenia, schizophrenias
#14	Schizophrenic Disorders
#15	Schizophrenic Disorder
#16	Disorder, Schizophrenic
#17	Disorders, Schizophrenic
#18	#13 OR #14 OR #15 OR #16 OR #17
#20	#12 AND #18

For peer review only

Searching other resources

The reference lists of published relevant academic journals and review articles will also be manually searched to identify any missing literature. Furthermore, clinical guidelines pertaining to the relevant trials and articles of an ambiguous nature will be manually scrutinized to ensure no eligible studies are overlooked.

Projected timeline for the review

The systematic review is slated to commence in January 2024 and is envisioned to reach

completion by June 2024.

Data collection

Selection of studies

Two independent researchers will independently review the titles, abstracts, and full texts of the articles retrieved, compiling studies that meet the inclusion criteria. Any disagreements between researchers will be resolved through discussion to reach a consensus, and if necessary, a third independent reviewer will contribute to the final decision. The flowchart of the Preferred Reporting Items for Systematic Reviews and Meta-Analyses (PRISMA) delineates the selection process of the studies (Figure 1).

Data extraction and management

Two researchers will independently extract the following information from the included articles using a standardized form: (1) Basic information: first author's name, year of publication, region of publication, number of cases included, type of study design; (2) Clinical and pathological data: age, gender, duration of illness, length of hospital stay, inpatient environment; (3) Intervention measures: nature of the intervention, implementer, frequency of intervention per week, duration of the intervention; (4) Outcome measures; (5) Rating scales used. Both researchers will independently extract and code data according to this form. The results of the data extraction will be cross-checked. In case of discrepancies, a third researcher will be involved in the discussion and decision-making process.

Assessment of risk of bias

The risk of bias and methodological quality of all included randomized controlled trials will be assessed by two independent researchers using the Cochrane Risk of Bias 2 (RoB 2) tool (43). Each trial will be evaluated for bias according to the following domains: bias arising from the randomization process, bias due to deviations from the intended interventions, bias due to missing outcome data, bias in measurement of the outcome, and bias in selection of the reported result. Risks of bias will be classified as

low, high, or unclear. In cases of disagreement, a third reviewer will be consulted, and consensus will be reached through discussion.

Data analysis

Measures of treatment effect

Review Manager (RevMan) 5.4 will be utilized to perform the meta-analysis. Continuous data will be expressed as mean difference (MD) and dichotomous data as risk ratio (RR), both with 95% confidence intervals (CI). When the same outcome is measured in various ways, the standardized mean difference (SMD) with a 95% CI will be chosen to represent continuous data.

Unit of analysis issues

The primary unit of analysis will be all individuals included in the control and experimental groups. Units of outcome measures from different studies will be converted to the International System of Units prior to analysis.

Dealing with missing data

Researchers will attempt to contact the authors included in the study via email to obtain missing or incomplete data. If missing data cannot be obtained, the study will be excluded.

Assessment of heterogeneity

Statistical heterogeneity among the included studies will be assessed using the I^2 statistic and the Chi^2 test. Studies will be considered heterogeneous when $I^2 > 50\%$ or $p < 0.1$.

Assessment of reporting biases

Reporting bias will be assessed following the CONSORT guidelines, and a funnel plot will be generated to evaluate potential reporting biases. A symmetric distribution of data within the funnel plot suggests the absence of publication bias. If the funnel plot

is asymmetrical, efforts will be made to explore possible explanations beyond publication biases and language biases.

Data synthesis

Data synthesis will be conducted using RevMan 5.4. Results from two or more heterogeneous studies will be combined using a random effects model ($I^2 > 50\%$ or $p < 0.1$). In the event that the meta-analysis is impeded by considerable clinical and methodological heterogeneity, a qualitative summary of the results will be provided.

Subgroup analysis and investigation of heterogeneity

In the presence of significant heterogeneity among the included studies, subgroup analyses will be conducted. Subgroups will be formed based on different populations (for example, male versus female), different types of GATs mediums (such as group painting, group music, or group play), and different art therapy settings (such as inpatient, outpatient, or community). The effect estimates for each subgroup will be calculated and compared inter-subgroup.

Sensitivity analysis

To ensure the robustness of the evidence, sensitivity analyses will be performed to assess the impact of studies with a high risk of bias and to explore possible sources of heterogeneity. Decisions to exclude lower-quality studies will be made by comparing outcomes and considering factors such as sample size, strength of evidence, and their influence on the pooled effective sample size.

Evidence quality evaluation

The quality of evidence will be further assessed using the Grading of Recommendations Assessment, Development and Evaluation (GRADE) approach, which categorizes the quality of evidence into four levels: very low, low, moderate, or high. In interpreting the findings, consideration will also be given to the quality of evidence, the context of the studies, potential benefits and harms, and patient values.

Patient and public involvement

Patients or the public were not involved in the study.

Ethics and dissemination

This systematic review does not require ethical approval, as all data utilized are anonymous and do not involve any private information. The outcomes of the research will offer evidence on the efficacy and safety of GATs as an adjunctive therapeutic approach for schizophrenia and will be disseminated through peer-reviewed publications.

Modifications to the preliminary proposal

Three significant modifications were made to the original protocol in response to reviewers' feedback. Firstly, a clear definition for GATs, based on the BAAT definitions, was provided to ensure alignment of the reviewed interventions with standard professional guidelines. Secondly, the criteria for the studies to be included in this review were detailed further. The revised standards now encompass a broader array of art therapies modalities such as painting, music, dance, yoga, drama, handicrafts, collage, play, photography, and movie. Moreover, the inclusion criteria were expanded to accommodate studies carried out exclusively in community settings. Lastly, recognizing the inherent variability in study designs and participant demographics within GATs, the expectation of low between-study heterogeneity may be overly optimistic. As such, the research methods clearly articulate how the statistical heterogeneity of the included studies was evaluated using the I^2 statistic and the Chi^2 test.

Discussion

While some explorations of GATs in the field of schizophrenia have been undertaken, evidence of its effectiveness in ameliorating positive, negative, and other related symptoms in patients remains limited. The inaugural MATISSE study on GATs indicated that GATs does not improve the overall functioning or mental health of individuals with schizophrenia (44), casting doubt on the overall efficacy of GATs.

Since then, researchers have been seeking evidence to support the mechanistic changes induced by GATs. Evidence from randomized controlled trials of group painting therapy (45), group music therapy (46), group play therapy (47), and group dance therapy (48) indicate that group forms of treatment using different art media can effectively improve a variety of clinical outcomes in patients with schizophrenia. Additionally, a large-scale study is examining GATs' effectiveness in a diagnostically diverse patient population within mental health services (20), yielding promising initial findings. However, these investigations have not systematically examined the efficacy of different GAT modalities.

A review study on arts therapies for schizophrenia was conducted in 2005 (49). But due to the early start of the study, only two randomized controlled trials with insufficient participants were included, making the results less meaningful. A meta-analysis initiated in July 2021 intended to assess arts therapy effectiveness for schizophrenia failed to report outcomes. Uttley et al.'s systematic review highlighted potential cost-effectiveness of GATs for non-psychotic disorders, yet noted the evidence was too limited for a definitive comparison (50). Reviews on drama therapy (51), dance therapy (52), and music therapy (53) examined the benefits and harms of different types of arts therapies for patient with schizophrenia. However, similar to previous studies, there is only moderate to low-quality evidence that arts therapies can be used as a supplement to standard care to improve relevant clinical outcomes in schizophrenia. At the same time, these efforts are not conducted in groups or small groups. Whether subgroups such as type, medium, duration, or frequency of GATs are related to treatment effectiveness remains unknown. The inclusion of large-scale and high-quality evidence and subgroup analyzes based on these variables may help address this issue.

This study aims to systematically assess the effectiveness of group-based artistic interventions in clinical treatment for schizophrenia, offering insights not provided by prior reviews. By examining a broader range of studies, including those published in English, Chinese, and Korean, this review seeks to enhance clinical evidence for healthcare providers and patients considering GATs as supplementary schizophrenia treatment.

Potential limitations of this study include possible language bias from excluding research in languages other than English, Chinese, and Korean, and publication bias due to the omission of conference papers, unpublished literature, and ongoing trials. Additionally, variability in GATs' types, mediums, durations, and frequencies may introduce heterogeneity. Nonetheless, this systematic approach still contributes to a broader understanding of GATs for schizophrenia.

Contributorship statement

AJZ wrote the draft of the manuscript, XXL and JW did independent reviews, RQL did research the reference. CHH, AJZ and RQL contributed to data organization, and JW and GHH critically reviewed the final draft of the manuscript. All authors contributed to the article and approved the submitted version.

Competing interests

The authors declared no conflict (competing) of interest.

Funding

This study is supported by the Research on Digital Art and Cultural Industry Development project of Guangzhou Wanqu Cooperative Institute of Design (9028) and Macau University of Science and Technology's Faculty Research Grant (No.: FRG-24-030-FA).

Figure Legends

Figure1. PRISMA flow diagram of the study process.

Table Legends

Table 1. Search strategies.

Appendix Legends

For peer review only - http://bmjopen.bmj.com/site/about/guidelines.xhtml

Appendix 1. Full search strategies for all databases.

References

1. Correll, C. U., Solmi, M., Croatto, G., Schneider, L. K., Rohani‐Montez, S. C., Fairley, L., ... & Tiihonen, J. (2022). Mortality in people with schizophrenia: a systematic review and meta‐analysis of relative risk and aggravating or attenuating factors. World Psychiatry, 21(2), 248-271. doi: 10.1002/wps.20994

2. Crespo-Facorro, B., Such, P., Nylander, A. G., Madera, J., Resemann, H. K., Worthington, E., ... & Newton, R. (2021). The burden of disease in early schizophrenia–a systematic literature review. Current Medical Research and Opinion, 37(1), 109-121. doi: 10.1080/03007995.2020.1841618

3. Andreasen, N. (1995). Symptoms, signs, and diagnosis of schizophrenia. The Lancet, 346(8973), 477-481. doi: 10.1016/S0140-6736(95)91325-4

4. Temmingh, H., & Stein, D. J. (2015). Anxiety in patients with schizophrenia: Epidemiology and management. CNS drugs, 29, 819-832. doi: 10.1007/s40263-015-0282-7

5. Marder, S. R., & Galderisi, S. (2017). The current conceptualization of negative symptoms in schizophrenia. World Psychiatry, 16(1), 14-24. doi: 10.1002/wps.20385

6. Buchanan, R. W. (2007). Persistent negative symptoms in schizophrenia: an overview. Schizophrenia bulletin, 33(4), 1013-1022. doi: 10.1093/schbul/sbl057

7. Milev, P., Ho, B. C., Arndt, S., & Andreasen, N. C. (2005). Predictive values of neurocognition and negative symptoms on functional outcome in schizophrenia: a longitudinal first-episode study with 7-year follow-up. American Journal of Psychiatry, 162(3), 495-506. doi: 10.1176/appi.ajp.162.3.495

8. Leucht, S., Tardy, M., Komossa, K., Heres, S., Kissling, W., Salanti, G., & Davis, J. M. (2012). Antipsychotic drugs versus placebo for relapse prevention in schizophrenia: a systematic review and meta-analysis. The Lancet, 379(9831), 2063-2071. doi: 10.1016/S0140-6736（12）60239-6

9. Kane, J. M., & Correll, C. U. (2010). Past and present progress in the pharmacologic treatment of schizophrenia. The Journal of clinical psychiatry, 71(9), 13909. doi：10.4088/JCP.10r06264yel

10. Kirkpatrick, B., Fenton, W. S., Carpenter Jr, W. T., & Marder, S. R. (2006). The NIMH-MATRICS consensus statement on negative symptoms. Schizophrenia bulletin, 32(2), 214-219. doi: 10.1093/schbul/sbj053

11. De las Cuevas, C., Sanz, E. J., & de Leon, J. (2023). Adverse drug reactions and their fatal outcomes in clozapine patients in VigiBase: Comparing the top four reporting countries (US, UK, Canada and Australia). Schizophrenia Research. doi: 10.1016/j.schres.2023.05.004

12. Papola, D., Ostuzzi, G., Gastaldon, C., Morgano, G. P., Dragioti, E., Carvalho, A. F., ... & Barbui, C. (2019). Antipsychotic use and risk of life - threatening medical events: umbrella review of observational studies. Acta Psychiatrica Scandinavica, 140(3), 227-243. doi: 10.1111/acps.13066

13. Lindström, E., & Bingefors, K. (2000). Patient compliance with drug therapy in schizophrenia: economic and clinical issues. Pharmacoeconomics, 18, 105-124. doi: 10.2165/00019053-200018020-00002

14. Peng, M. M., Xing, J., Tang, X., Wu, Q., Wei, D., & Ran, M. S. (2022). Disease-related risk factors for caregiver burden among family caregivers of persons with schizophrenia: a systematic review and meta-analysis. International Journal of Environmental Research and Public Health, 19(3), 1862. doi: 10.3390/ijerph19031862

15. Jin, H., & Mosweu, I. (2017). The societal cost of schizophrenia: a systematic review. Pharmacoeconomics, 35, 25-42. doi: 10.1007/s40273-016-0444-6

16. Moon, B. L. (2016). Art-based group therapy: Theory and practice. Charles C Thomas Publisher.

17. Liebmann, M. (2004). Art therapy for groups: A handbook of themes and exercises. Psychology Press.

18. Hui, H. (2023). Intervention Study of Expressive Art Group Therapy on Children with Emotional Distress. Advances in Psychology, 13(7), 2757-2765. https://doi.org/10.12677/AP.2023.137340

19. American Group Psychotherapy Association Science to Service Task Force. Practice guidelines for group psychotherapy. American Group Psychotherapy Association; 2007.

20. Carr, C. E., Medlicott, E., Hooper, R., Feng, Y., Mihaylova, B., & Priebe, S. (2023). Effectiveness of group arts therapies (art therapy, dance movement therapy and music therapy) compared to group counselling for diagnostically heterogeneous psychiatric community patients: study protocol for a randomised controlled trial in mental health services (the ERA study). Trials, 24(1), 557. https://doi.org/10.1186/s13063-023-07232-0

21. Karkou V, Sanderson P. (2005). Arts therapies: a research-based map of the field. London: Elsevier Churchill Livingstone.

22. Orfanos, S., Banks, C., & Priebe, S. (2015). Are group psychotherapeutic treatments effective for patients with schizophrenia? A systematic review and meta-analysis. Psychotherapy and psychosomatics, 84(4), 241-249. https://doi.org/10.1159/000377705

23. Cho, J. M., & Lee, K. (2018). Effects of motivation interviewing using a group art therapy program on negative symptoms of schizophrenia. Archives of psychiatric nursing, 32(6), 878-884. https://doi.org/10.1016/j.apnu.2018.07.002

24. Tong, J., Yu, W., Fan, X., Sun, X., Zhang, J., Zhang, J., & Zhang, T. (2021). Impact of group art therapy using traditional Chinese materials on self-efficacy and social function for individuals diagnosed with schizophrenia. Frontiers in Psychology, 11, 571124. https://doi.org/10.3389/fpsyg.2020.571124

25. Green, B. L., Wehling, C., & Taisky, G. J. (1987). Group art therapy as an adjunct to treatment for chronic outpatients. Psychiatric Services, 38(9), 988-991. https://doi.org/10.1176/ps.38.9.988

26. Waller, D. (2014). Group interactive art therapy: Its use in training and treatment. Routledge.

27. Chandraiah, S., Ainlay Anand, S., & Avent, L. C. (2012). Efficacy of group art therapy on depressive symptoms in adult heterogeneous psychiatric outpatients. Art Therapy, 29(2), 80-86. doi: 10.1080/07421656.2012.683739

28. Waller, D., & Gilroy, A. (2000). Art Therapy: A handbook of new theories and practice. Buckingham: Open University Press.

29. Gabel, A., & Robb, M. (2017). (Re) considering psychological constructs: A thematic synthesis defining five therapeutic factors in group art therapy. The arts in psychotherapy, 55, 126-135. doi: 10.1016/j.aip.2017.05.005

30. Jewell, M., & Camden-Pratt, C. (2023). Creating 'art-alongside'in Peer Art Therapy (PATh) groups: nurturing connection and trust, and responding to power dynamics. International Journal of Art Therapy, 28:1-2, 38-50. doi: 10.1080/17454832.2023.2175002

31. Springham, N., & Camic, P. M. (2017). Observing mentalizing art therapy groups for people diagnosed with borderline personality disorder. International Journal of Art Therapy, 22(3), 138-152. https://doi.org/10.1080/17454832.2017.1288753

32. Rainbow T. H. Ho, Jordan S. Potash, Andy H. Y. Ho, Vania F. L. Ho & Eric Y. H. Chen. (2017). Reducing mental illness stigma and fostering empathic citizenship: Community arts collaborative approach, Social Work in Mental Health, 15:4, 469-485. DOI: 10.1080/15332985.2016.1236767

33. Kapitan, L., Litell, M., & Torres, A. (2011). Creative art therapy in a community's participatory research and social transformation. Art Therapy, 28(2), 64-73. DOI: 10.1080/07421656.2011.578238

34. Oren, R., Orkibi, H., Elefant, C., & Salomon-Gimmon, M. (2019). Arts-based psychiatric rehabilitation programs in the community: Perceptions of healthcare professionals. Psychiatric Rehabilitation Journal, 42(1), 41–47. https://doi.org/10.1037/prj0000325

35. Armijo, J., Méndez, E., Morales, R., Schilling, S., Castro, A., Alvarado, R., & Rojas, G. (2013). Efficacy of community treatments for schizophrenia and other psychotic disorders: a literature review. Frontiers in psychiatry, 4, 116. https://doi.org/10.3389/fpsyt.2013.00116

36. Spitzer, R. L., Williams, J. B., & Skodol, A. E. (1980). DSM-III: the major achievements and an overview. The American Journal of Psychiatry, 137(2), 151-164. https://doi.org/10.1176/ajp.137.2.151

37. Widiger, T. A., Frances, A., Spitzer, R. L., & Williams, J. B. (1988). The DSM-III-R personality disorders: an overview. The American Journal of Psychiatry, 145(7), 786-795. https://doi.org/10.1176/ajp.145.7.786

38. Frances, A., First, M. B., & Pincus, H. A. (1995). DSM-IV guidebook. American Psychiatric Association.

39. World Health Organization. (1992). The ICD-10 classification of mental and behavioural disorders: clinical descriptions and diagnostic guidelines (Vol. 1). World Health Organization.

40. Edwards D. (2004). Art therapy. Sage.

41. Gowda, G. S., & Isaac, M. K. (2022). Models of care of schizophrenia in the community—an international perspective. Current psychiatry reports, 24(3), 195-202. https://doi.org/10.1007/s11920-022-01329-0

42. Lim, C., Barrio, C., Hernandez, M., Barragán, A., Yamada, A.-M., & Brekke, J. S. (2016). Remission of symptoms in community-based psychosocial rehabilitation services for individuals with schizophrenia. Psychiatric Rehabilitation Journal, 39(1), 42–46. https://doi.org/10.1037/prj0000154

43. Higgins, J. P., Sterne, J. A., Savovic, J., Page, M. J., Hróbjartsson, A., Boutron, I., ... & Eldridge, S. (2016). A revised tool for assessing risk of bias in randomized trials. Cochrane database of systematic reviews, 10 (Suppl 1), 29-31. dx.doi.org/10.1002/14651858.CD201601

44. Crawford, M. J., Killaspy, H., Kalaitzaki, E., Barrett, B., Byford, S., Patterson, S., ... & Waller, D. (2010). The MATISSE study: a randomised trial of group art

455 therapy for people with schizophrenia. BMC psychiatry, 10(1), 1-9.
456 https://doi.org/10.1186/1471-244X-10-65

457 45. Cho, J. M., & Lee, K. (2018). Effects of motivation interviewing using a group art
458 therapy program on negative symptoms of schizophrenia. Archives of psychiatric
459 nursing, 32(6), 878-884. https://doi.org/10.1016/j.apnu.2018.07.002

460 46. Peng, S. M., Koo, M., & Kuo, J. C. (2010). Effect of group music activity as an
461 adjunctive therapy on psychotic symptoms in patients with acute schizophrenia.
462 Archives of psychiatric nursing, 24(6), 429-434.
463 https://doi.org/10.1016/j.apnu.2010.04.001

464 47. Torres, A., Mendez, L. P., Merino, H., & Moran, E. A. (2002). Rehab rounds:
465 Improving social functioning in schizophrenia by playing the train game.
466 Psychiatric Services, 53(7), 799-801. https://doi.org/10.1176/appi.ps.53.7.799

467 48. Bryl, K., Bradt, J., Cechnicki, A., Fisher, K., Sossin, K. M., & Goodill, S. (2022).
468 The role of dance/movement therapy in the treatment of negative symptoms in
469 schizophrenia: a mixed methods pilot study. Journal of Mental Health, 31(5), 613-
470 623. https://doi.org/10.1080/09638237.2020.1757051

471 49. Ruddy, R., & Milnes, D. (2005). Art therapy for schizophrenia or schizophrenia
472 ‐ like illnesses. Cochrane Database of Systematic Reviews, (4).
473 https://doi.org/10.1002/14651858.CD003728.pub2

474 50. Uttley, L., Stevenson, M., Scope, A., Rawdin, A., & Sutton, A. (2015). The clinical
475 and cost effectiveness of group art therapy for people with non-psychotic mental
476 health disorders: a systematic review and cost-effectiveness analysis. BMC
477 psychiatry, 15(1), 1-13. https://doi.org/10.1186/s12888-015-0528-4

478 51. Ruddy, R., & Dent ‐ Brown, K. (2007). Drama therapy for schizophrenia or
479 schizophrenia ‐ like illnesses. Cochrane Database of Systematic Reviews, (1).
480 https://doi.org/10.1002/14651858.CD005378.pub2

481 52. Ren, J., & Xia, J. (2013). Dance therapy for schizophrenia. Cochrane Database of
482 Systematic Reviews, (10). https://doi.org/10.1002/14651858.CD006868.pub3

483 53. Geretsegger, M., Mössler, K. A., Bieleninik, Ł., Chen, X. J., Heldal, T. O., & Gold,
484 C. (2017). Music therapy for people with schizophrenia and schizophrenia ‐ like
485 disorders. Cochrane Database of Systematic Reviews, (5).
486 https://doi.org/10.1002/14651858.CD004025.pub4

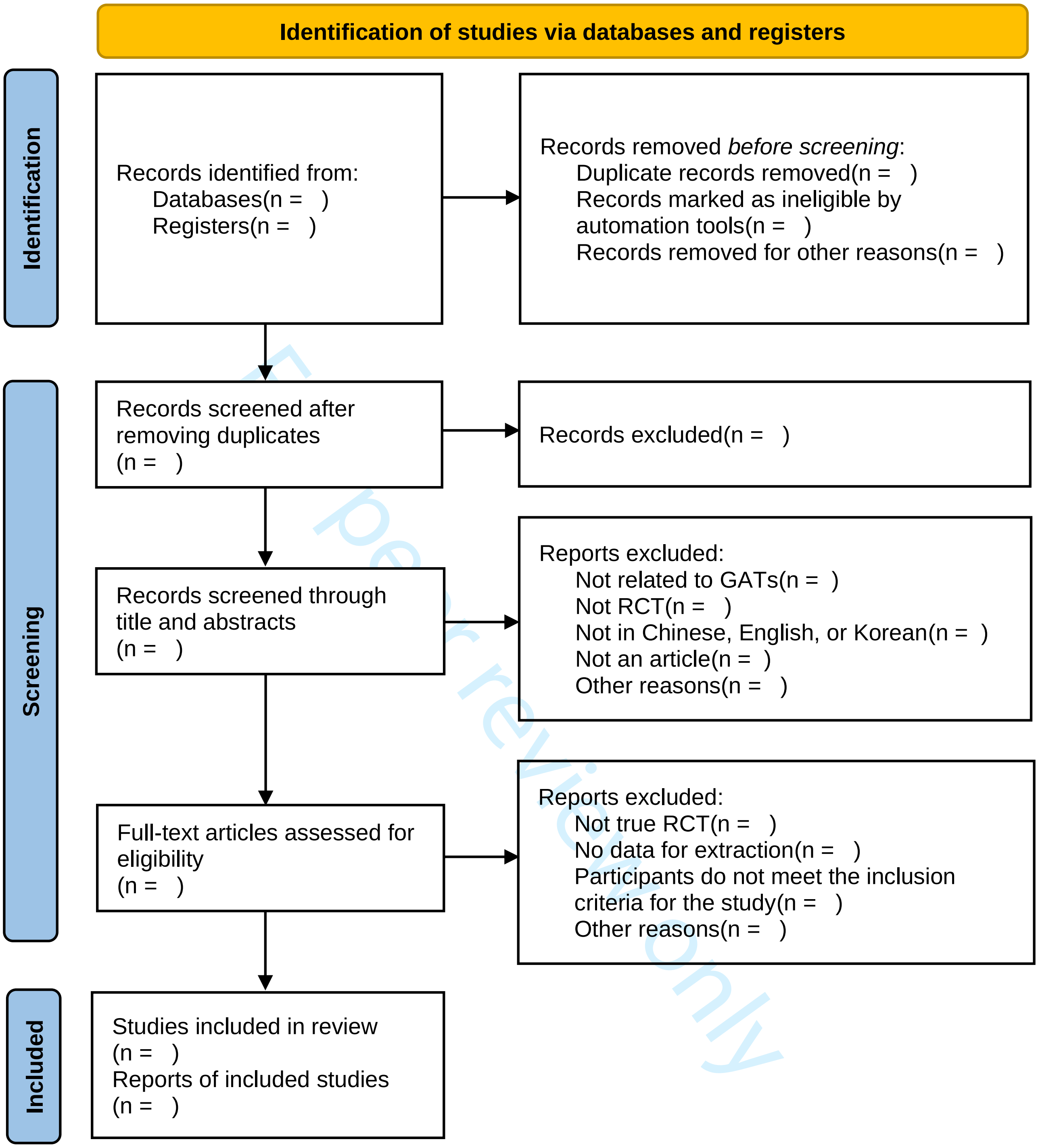

Figure 1. PRISMA flow diagram of the study process. PRISMA, Preferred Reporting Items for Systematic review and Meta-Analysis; RCT, randomized controlled trial; GATs, group arts therapies.

For peer review only

Group Arts Therapies for Patients with Schizophrenia: A Protocol of Systematic Review and Meta-analysis

Appendix 1. Full search strategies for all databases.

Database	Search strategy
PubMed	(((((((((((((((group arts therapies[Title/Abstract]) OR (group painting therapy[Title/Abstract])) OR (group music therapy[Title/Abstract])) OR (group dance therapy[Title/Abstract])) OR (group yoga therapy[Title/Abstract])) OR (group drama therapy[Title/Abstract])) OR (group handicrafts therapy[Title/Abstract])) OR (group collage therapy[Title/Abstract])) OR (group play therapy[Title/Abstract])) OR (group photography therapy[Title/Abstract])) OR (group movie therapy[Title/Abstract])) AND (Schizophrenia, schizophrenias[Title/Abstract])) OR (Schizophrenic Disorders[Title/Abstract])) OR (Schizophrenic Disorder[Title/Abstract])) OR (Disorder, Schizophrenic[Title/Abstract])) OR (Disorders, Schizophrenic[Title/Abstract])
Web of Science	(ALL=(Group Arts therapies)) OR ALL=(Group Painting Therapy)) OR ALL=(Group Music Therapy)) OR ALL=(Group Dance Therapy)) OR ALL=(Group Yoga Therapy)) OR ALL=(Group Drama Therapy)) OR ALL=(Group Handicrafts Therapy)) OR ALL=(Group Collage Therapy)) OR ALL=(Group Play Therapy)) OR ALL=(Group Photography Therapy)) OR ALL=(Group Movie Therapy)) AND ALL=(Schizophrenia, schizophrenias)) OR ALL=(Schizophrenic Disorders)) OR ALL=(Schizophrenic Disorder)) OR ALL=(Disorder, Schizophrenic)) OR ALL=(Disorders, Schizophrenic)
Cochrane Library	Number — Search terms<br>#1 Group Arts therapies<br>#2 Group Painting Therapy<br>#3 Group Music Therapy<br>#4 Group Dance Therapy<br>#5 Group Yoga Therapy<br>#6 Group Drama Therapy<br>#7 Group Handicrafts Therapy<br>#8 Group Collage Therapy<br>#9 Group Play Therapy<br>#10 Group Photography Therapy<br>#11 Group Movie Therapy<br>#12 #1 OR #2 OR #3 OR #4 OR #5 OR #6 OR #7 OR #8 OR #9 OR #10 OR #11<br>#13 Schizophrenia, schizophrenias<br>#14 Schizophrenic Disorders<br>#15 Schizophrenic Disorder<br>#16 Disorder, Schizophrenic<br>#17 Disorders, Schizophrenic<br>#18 #13 OR #14 OR #15 OR #16 OR #17<br>#20 #12 AND #18
Embase	('group arts therapies' OR (('group'/exp OR group) AND arts AND therapies) OR 'group painting therapy' OR (('group'/exp OR group) AND

For peer review

	('painting'/exp OR painting) AND ('therapy'/exp OR therapy)) OR 'group music therapy' OR (('group'/exp OR group) AND ('music'/exp OR music) AND ('therapy'/exp OR therapy)) OR 'group dance therapy' OR (('group'/exp OR group) AND ('dance'/exp OR dance) AND ('therapy'/exp OR therapy)) OR 'group yoga therapy' OR (('group'/exp OR group) AND ('yoga'/exp OR yoga) AND ('therapy'/exp OR therapy)) OR 'group drama therapy' OR (('group'/exp OR group) AND ('drama'/exp OR drama) AND ('therapy'/exp OR therapy)) OR 'group handicrafts therapy' OR (('group'/exp OR group) AND handicrafts AND ('therapy'/exp OR therapy)) OR 'group collage therapy' OR (('group'/exp OR group) AND collage AND ('therapy'/exp OR therapy)) OR 'group play therapy' OR (('group'/exp OR group) AND ('play'/exp OR play) AND ('therapy'/exp OR therapy)) OR 'group photography therapy' OR (('group'/exp OR group) AND ('photography'/exp OR photography) AND ('therapy'/exp OR therapy)) OR 'group movie therapy' OR (('group'/exp OR group) AND ('movie'/exp OR movie) AND ('therapy'/exp OR therapy))) AND ('schizophrenia, schizophrenias' OR (('schizophrenia,'/exp OR schizophrenia,) AND schizophrenias) OR 'schizophrenic disorders' OR (('schizophrenic'/exp OR schizophrenic) AND ('disorders'/exp OR disorders)) OR 'schizophrenic disorder' OR (('schizophrenic'/exp OR schizophrenic) AND ('disorder'/exp OR disorder)) OR 'disorder, schizophrenic' OR (('disorder,'/exp OR disorder,) AND ('schizophrenic'/exp OR schizophrenic)) OR 'disorders, schizophrenic' OR (('disorders,'/exp OR disorders,) AND ('schizophrenic'/exp OR schizophrenic)))
China National Knowledge Infrastructure (CNKI)	（团体艺术治疗 OR 团体绘画疗法 OR 团体音乐疗法 OR 团体舞蹈疗法 OR 团体瑜伽疗法 OR 团体戏剧疗法 OR 团体手工艺疗法 OR 团体拼贴画疗法 OR 团体游戏疗法 OR 团体摄影疗法 OR 团体电影疗法） AND （精神分裂症 OR 精神分裂）
Wanfang Data	（团体艺术治疗 或 团体绘画疗法 或 团体音乐疗法 或 团体舞蹈疗法 或 团体瑜伽疗法 或 团体戏剧疗法 或 团体手工艺疗法 或 团体拼贴画疗法 或 团体游戏疗法 或 团体摄影疗法 或 团体电影疗法） 与 （精神分裂症 或 精神分裂）
RISS	(((((전체=(집단 예술 치료) OR 전체=(집단 미술 치료) OR 전체=(집단 음악 치료) OR 전체=(집단 무용 치료) OR 전체=(집단 요가 치료) OR 전체=(집단 드라마 치료) OR 전체=(집단 수공예 치료) OR 전체=(집단 콜라주 치료) OR 전체=(집단 놀이 치료) OR 전체=(집단 사진 치료) OR 전체=(집단 영화 치료)) AND 전체=(조현병)) OR 전체=(정신분열증))
Korean Citation Index (KCI)	(((((전체=(집단 예술 치료) OR 전체=(집단 미술 치료) OR 전체=(집단 음악 치료) OR 전체=(집단 무용 치료) OR 전체=(집단 요가 치료) OR 전체=(집단 드라마 치료) OR 전체=(집단 수공예 치료) OR 전체=(집단 콜라주 치료) OR 전체=(집단 놀이 치료) OR 전체=(집단 사진 치료) OR 전체=(집단 영화 치료)) AND 전체=(조현병)) OR 전체=(정신분열증))

For peer review only

DBpia	(((((전체=(집단 예술 치료) OR 전체=(집단 미술 치료) OR 전체=(집단 음악 치료) OR 전체=(집단 무용 치료) OR 전체=(집단 요가 치료) OR 전체=(집단 드라마 치료) OR 전체=(집단 수공예 치료) OR 전체=(집단 콜라주 치료) OR 전체=(집단 놀이 치료) OR 전체=(집단 사진 치료) OR 전체=(집단 영화 치료)) AND 전체=(조현병)) OR 전체=(정신분열증))

For peer review only

PRISMA-P (Preferred Reporting Items for Systematic review and Meta-Analysis Protocols) 2015 checklist: recommended items to address in a systematic review protocol*

	Section and topic	Item No	Checklist item
	**ADMINISTRATIVE INFORMATION**		
1-2	Title:		
	Identification	1a	Identify the report as a protocol of a systematic review
	Update	1b	If the protocol is for an update of a previous systematic review, identify as such
29	Registration	2	If registered, provide the name of the registry (such as PROSPERO) and registration number
Anonymization of submission documents	Authors:		
	Contact	3a	Provide name, institutional affiliation, e-mail address of all protocol authors; provide physical mailing address of corresponding author
	Contributions	3b	Describe contributions of protocol authors and identify the guarantor of the review
/	Amendments	4	If the protocol represents an amendment of a previously completed or published protocol, identify as such and list changes; otherwise, state plan for documenting important protocol amendments
304-308	Support:		
	Sources	5a	Indicate sources of financial or other support for the review
	Sponsor	5b	Provide name for the review funder and/or sponsor
	Role of sponsor or funder	5c	Describe roles of funder(s), sponsor(s), and/or institution(s), if any, in developing the protocol
	**INTRODUCTION**		
42-81	Rationale	6	Describe the rationale for the review in the context of what is already known
82-85	Objectives	7	Provide an explicit statement of the question(s) the review will address with reference to participants, interventions, comparators, and outcomes (PICO)
	**METHODS**		
88-155	Eligibility criteria	8	Specify the study characteristics (such as PICO, study design, setting, time frame) and report characteristics (such as years considered, language, publication status) to be used as criteria for eligibility for the review
139-155	Information sources	9	Describe all intended information sources (such as electronic databases, contact with study authors, trial registers or other grey literature sources) with planned dates of coverage
table 1 appendix 1	Search strategy	10	Present draft of search strategy to be used for at least one electronic database, including planned limits, such that it could be repeated
	Study records:		
164-174	Data management	11a	Describe the mechanism(s) that will be used to manage records and data throughout the review

For peer review only

157-163	Selection process	11b	State the process that will be used for selecting studies (such as two independent reviewers) through each phase of the review (that is, screening, eligibility and inclusion in meta-analysis)
165-174	Data collection process	11c	Describe planned method of extracting data from reports (such as piloting forms, done independently, in duplicate), any processes for obtaining and confirming data from investigators
165-174	Data items	12	List and define all variables for which data will be sought (such as PICO items, funding sources), any pre-planned data assumptions and simplifications
127-138	Outcomes and prioritization	13	List and define all outcomes for which data will be sought, including prioritization of main and additional outcomes, with rationale
175-183	Risk of bias in individual studies	14	Describe anticipated methods for assessing risk of bias of individual studies, including whether this will be done at the outcome or study level, or both; state how this information will be used in data synthesis
209-213	Data synthesis	15a	Describe criteria under which study data will be quantitatively synthesised
		15b	If data are appropriate for quantitative synthesis, describe planned summary measures, methods of handling data and methods of combining data from studies, including any planned exploration of consistency (such as I^2, Kendall's τ)
		15c	Describe any proposed additional analyses (such as sensitivity or subgroup analyses, meta-regression)
		15d	If quantitative synthesis is not appropriate, describe the type of summary planned
204-208 and 222-226	Meta-bias(es)	16	Specify any planned assessment of meta-bias(es) (such as publication bias across studies, selective reporting within studies)
228-232	Confidence in cumulative evidence	17	Describe how the strength of the body of evidence will be assessed (such as GRADE)

*** It is strongly recommended that this checklist be read in conjunction with the PRISMA-P Explanation and Elaboration (cite when available) for important clarification on the items. Amendments to a review protocol should be tracked and dated. The copyright for PRISMA-P (including checklist) is held by the PRISMA-P Group and is distributed under a Creative Commons Attribution Licence 4.0.**

From: Shamseer L, Moher D, Clarke M, Ghersi D, Liberati A, Petticrew M, Shekelle P, Stewart L, PRISMA-P Group. Preferred reporting items for systematic review and meta-analysis protocols (PRISMA-P) 2015: elaboration and explanation. BMJ. 2015 Jan 2;349(jan02 1):g7647.

For peer review only
